# The Terminal Diner: Serving up a Novel Knowledge Exchange Methodology via Participatory Design Installation

**DOI:** 10.1111/hex.70688

**Published:** 2026-06-23

**Authors:** Aria Wills, Kate Wilkes, Karen Oikonen, Nyanna Flynn, Shuaib Hafid, Aleisha Fernandes, Michelle Howard, Sarina R. Isenberg

**Affiliations:** ^1^ Bruyère Health Research Institute Ottawa Ontario Canada; ^2^ Independent Scholar Ottawa Ontario Canada; ^3^ The Moment Ottawa Ontario Canada; ^4^ Caregiver Advisor Ottawa Ontario Canada; ^5^ Department of Family Medicine McMaster University Hamilton Ontario Canada; ^6^ Department of Health Research Methods Evidence, and Impact, McMaster University Hamilton Ontario Canada; ^7^ Ottawa Hospital Research Institute Ottawa Ontario Canada; ^8^ Department of Medicine, Faculty of Medicine University of Ottawa Ottawa Ontario Canada

**Keywords:** arts‐based knowledge mobilisation, co‐design, continuity of care, end of life, knowledge exchange, participatory design, public engagement

## Abstract

**Context:**

Arts‐based knowledge mobilisation (ABKM) is a powerful means of interpreting and communicating health services research, centreing experiential, iterative, and reflective knowledge rooted in co‐creation. We developed a *research‐to‐public‐to‐research* feedback loop methodology extending beyond existing ABKM practices and our previous *research‐to‐public* methodology, creating a novel bidirectional epistemic process wherein meaning making occurs from research to public and from public to research. This approach produces knowledge that is rich, contextually grounded, and otherwise potentially inaccessible through conventional research.

**Objectives:**

We present a case study documenting the first application of the feedback loop, with the aim of advancing and refining the methodological framework by: sharing quantitative findings with the public and eliciting public engagement via a design installation, and levering engagement to inform subsequent qualitative research.

**Methods:**

Using the design‐driven *research‐to‐public‐to‐research* feedback loop, we developed a participatory design installation focused on end‐of‐life care. Within an overarching mixed‐methods sequential study, we leveraged design and health services methods to: develop design principles; select installation aims and concept; prototype, refine, and iterate the design; and conduct descriptive analysis of installation outputs.

**Results:**

Borrowing characteristics of a classic diner, the installation entitled *The Terminal Diner*, invited individuals to complete orders for their desired end‐of‐life healthcare experience or leave reviews drawn from lived experience with a deceased loved one's end‐of‐life journey. The installation disseminated quantitative findings and engaged the general public in contemplating and co‐creating around the subject of death—outputs of which were used to inform subsequent qualitative research, while recruiting an interested audience as research participants.

**Conclusion:**

This study presents an innovative extension of our previous *research‐to‐public* methodology, representing a paradigmatic shift towards creative knowledge exchange and bidirectional meaning making within health services research.

**Patient Public Contribution:**

The public's role as co‐creators is foundational to the *research‐to‐public‐to‐research* feedback loop methodology. Those with lived experience—bereaved caregivers and patient partners—were also embedded throughout both the research and design processes, ensuring accessibility, relevance, and emotional resonance. Blending personal knowledge and public perspective, *The Terminal Diner* foregrounded the essential role of patient and public contributions in co‐creating meaningful, responsive, and inclusive health services research.

## 1 Introduction

There exists a growing effort to share findings from health services research beyond the confines of academia. The movement challenges and subverts conventional means of knowledge exchange, as such empirical approaches often fail to capture the experiential and existential facets of complex or emotionally sensitive areas of research, such as end‐of‐life (EOL) care [1, 2, 3].

Arts‐based knowledge mobilisation (ABKM), which encompasses design‐driven practice, has emerged as a powerful means of interpreting and communicating health services research [2, 4]. Underpinning ABKM is a set of foundational philosophical concepts that shape its methodology and offer unique ways of knowing, making this approach especially well‐suited and valuable for EOL research [5]. First, ABKM is grounded in practice‐enabled knowledge, which foregrounds experiential ways of knowing generated through creative engagement [1, 6, 7]. ABKM also draws on pragmatic enquiry, which sees the process of knowing as inherently iterative and embedded in lived experience [8, 9, 10, 11]. Finally, ABKM positions knowledge as something developed through reflection‐in‐action and reflection‐on‐action, where knowledge is dynamic and responsive to real, ill‐defined problems [12, 13, 14]. The ABKM approach thus centres the kinds of experiential, iterative, and reflective understanding, and sees them as inseparable from creative engagement through which knowledge is interpreted and disseminated.

ABKM also rests on the core value of co‐creation, and including individuals with lived experience throughout the creation process [15]; this embeds the belief that humans are inherently creative and experts of their own experiences. A nascent approach within ABKM is participatory design, wherein a design piece (which involves the development of a work or exhibit that can vary in form to creatively convey a concept) is only made whole and complete through participant engagement. In this way, participants actively construct meaning through ‘collective making’ [4]. This approach is rooted in doing, creating an embodied and practical form of knowing and acting as a medium of enquiry uniquely capable of making collective understanding from lived experience.

Leveraging these foundational underpinnings, we developed a *research‐to‐public‐to‐research* feedback loop, which represents an intersection between arts‐based research, experience‐based design research, and embodies a participatory ABKM approach [16, 17]. The feedback loop is an innovative strategy to engage the public throughout all stages of knowledge exchange—beginning with research, we disseminate conventional findings to the general public through art or design, and using those same creative means, gather the public's input to inform subsequent research. The *research‐to‐public* approach was previously developed and employed by our team in an EOL context to share research findings regarding transitioning from hospital to home at EOL and to elicit insights from the general public on these findings [16]. Combining existing ABKM means of dissemination with creative participatory practices, the feedback loop methodology represents a bidirectional epistemic process wherein meaning making occurs from research to public, and from public to research. This process extends most current ABKM practices, which are unidirectional in disseminating research through creative outputs or incorporating community input into design processes. For complex topics where nuanced, lived experience resists reduction to data, the feedback loop methodology produces knowledge that is rich, contextually grounded, and otherwise potentially inaccessible through conventional research [1, 2, 3].

We applied the *research‐to‐public‐to‐research* feedback loop methodology to examine continuity of care (COC) at the EOL through a multi‐year, multi‐method programme of research. COC comprises healthcare experienced as connected and consistent with patients' medical and personal needs [18], which was of particular interest due to its significant impact on patient experience and its role in enhancing overall care quality and satisfaction. Phase 1 (*research*) entailed three population‐based cohort studies of the COC experience at the EOL in patients with kidney failure on dialysis (KF‐D), advanced chronic obstructive pulmonary disease (COPD), and heart failure (HF) in Ontario (as described previously) [19, 20, 21]. Phase 2 (*public)* comprised the participatory installation, which was informed by the preceding Phase 1. Phase 3 (*research*) entailed qualitative interviews conducted with bereaved caregivers and informed by Phases 1 and 2 [22].

We present here a case study describing Phase 2 of the feedback loop, a public participatory design installation, and tracing the process of the methodology from Phase 1 to 3; in doing so, we have documented the *research‐to‐public‐to‐research* feedback loop process for the first time. This case study aimed to apply and refine the *research‐to‐public‐to‐research* feedback loop methodology by: (1) educating the public of our quantitative research findings using a creative method of knowledge dissemination; (2) gathering and analysing input collected via participatory design from the general public on their related EOL experiences; and (3) leveraging design to effectively recruit study participants and inform qualitative research.

## 2 Materials and Methods

*The Feedback Loop Methodology: Moving From Research to Public to Research*

We employed a design‐driven *research‐to‐public‐to‐research* feedback loop approach to elicit embodied meaning‐making regarding COC at the EOL—moving from the Phase 1 (*research)*, to develop a participatory design installation of Phase 2 *(public)*, which would inform Phase 3 (*research*). This process entailed iterative cycles of collaborative discussion and decision‐making regarding: contemplation of quantitative findings (Phase 1), and selection of the research problem and installation direction; development of design principles; installation concept ideation and selection; prototyping and refinement; iteration of key installation features between exhibitions; and translating insight gained through the design installation to inform subsequent qualitative research (Phase 3).

*Design Methodology*

Using a *research‐as‐design* approach, we created a participatory, public installation, for which the concept, form, and modalities (i.e., visual, auditory, and sensory) remained flexible and iterative [23]. Our constraints entailed creating a physical, transportable, interactive display which could be left unattended, eliciting unfacilitated, anonymous, and autonomous engagement.

In alignment with the gold standard for co‐design in palliative care [24], the project team worked collaboratively across all stages of the design, and comprised health services researchers (SI, MH, SH, AF, and AW), design researchers (KW and KO), and caregiver and patient partners (three caregivers, including NF, and one patient) with lived experience whose inclusion provided valuable insights into an end‐user perspective of engaging with the installation [23]. This approach ensured the incorporation of diverse perspectives throughout the design process while remaining mindful of our own biases and assumptions, to ensure the accessibility and responsivity of the design installation to the public and contexts in which it would be shown.

The team met virtually via Zoom (https://zoom.us) ten times from June 2023 to February 2024 (Supporting Information: File 1 describes the nature of these meetings). The design researchers employed Miro (https://www.miro.com), a shared virtual visual workspace, to engage the team in the design process.

### 2.1 Contemplation of Quantitative Findings and Selection of Installation Direction

During two meetings, the team reviewed key findings from the Phase 1 quantitative research presented by the health services researchers. Via three retrospective population‐based cohort studies using de‐identified health administrative data housed at ICES, an independent non‐profit research organisation in Ontario, this previous work examined the involvement of various physician specialties at the EOL across three disease populations—KF‐D, COPD, and HF—and identified patients' healthcare utilisation in relation to each pattern of physician involvement (results described elsewhere) [19, 20, 21]. Discussion centred around how to translate these findings to be engaging and educational for a broader audience. The design researchers posed probing questions to elucidate team members' perceptions of significant and compelling elements of the results for inclusion in the installation.

### 2.2 Development of Design Principles

The team met once to explore and deliberate on a variety of examples of participatory design installations, each with different aesthetic, conceptual, and participatory approaches. This deliberation enabled the team to identify key qualities to strive for and resulted in the articulation of the design principles. Design principles acted as ‘guardrails’—that is, a shared reference that the team could return to when making decisions about the installation's form, content, and means of interaction. Rather than prescribing specific outputs, the principles constrained the design space. This ensured that creative and technical decisions were aligned with the team's collective values and goals, while allowing for possibility, exploration, and creativity [25].

### 2.3 Concept Ideation, Selection, and Refinement

Design researchers incorporated the quantitative findings and design principles to present initial conceptual directions to the team. Iterations were developed over the course of three meetings through a cycle of feedback between design researchers and the team (health services researchers and a caregiver partner). For each concept, the team considered a description that included concept‐specific considerations, exemplar images of the means of engagement, and the design researchers' low‐fidelity concept sketch. The team provided feedback using the ‘Rose, Bud, Thorn’ structure (Rose: what is positive about this idea? Bud: what has potential or what could we build on? Thorn: what is negative or not working well?) and voted on overall preferences between concepts.

The design researchers combined compelling directions into unified concepts with refined probes and components. For each concept, they presented descriptions, examples of engagement mediums, material inspiration, and sketches illustrating their vision. The team provided their perspectives on perceived strengths, challenges, suggested adaptations, and overall preferences. Throughout this phase, we also planned the incorporation of a research participant recruitment element for subsequent qualitative interviews (Phase 3) focused on experiences of COC at EOL.

### 2.4 Installation Prototyping, Production, and Iteration

Drawing on the team's feedback, the design researchers developed a first‐iteration mock‐up of the installation, presenting an evolved concept that included structural form, interactive materials, and initial sketches. The team collaboratively developed content for the interactive materials. The design researchers then developed a low‐fidelity prototype. Prototyped interactive materials were presented to non‐team members, including friends and family of the team, to conduct a rapid, pragmatic check of the appropriateness and comprehensibility of the probe. Questions to elicit feedback included: “what is your overall feedback on the menu?”, “what is your understanding of the questions and options being asked of you?”, and “what is your understanding of the task that the exhibit viewer is being asked to do?”. Given the selective nature of this sample, feedback was used to ensure conceptual clarity, informing minor refinements rather than substantive design decisions.

The design researchers then developed high‐fidelity prototypes, graphically designing the installation's structure and interactive materials. Pre‐production entailed finalisation of the prototypes and printing of components. Production involved assembly of the installation's structure. Prototyping and production were coordinated and executed over the course of five meetings (definitions of design terms available in Giannitrapani et al. [24]).

#### 2.4.1 Iterations

*The Terminal Diner* was displayed at venues across the Canadian provinces of Ontario (ON), Quebec (QC), and British Columbia (BC), including Evergreen Brick Works (Toronto, ON), McMaster University's David Braley Health Sciences Centre (Hamilton, ON), Ottawa School of Art (Ottawa, ON), Bruyère Health's St. Vincent Hospital (Ottawa, ON), the McGill International Palliative Care Congress (Montreal, QC), the Champlain Hospice Palliative Care Program Education Day (Ottawa, ON), and the European Association for Palliative Care International Research Seminar on Public Health & Palliative Care (Victoria, BC).

Evergreen Brick Works was scouted and coordinated by a design researcher (KH). While the installation was created to be unfacilitated, team members (KW, KO and SI) were present on site for one day to informally observe and take unstructured field notes recording researchers' reflexive observations regarding participants' engagement with the installation. Based on discussion of the team's observations, we revised and reproduced the installation's elements and materials to enhance participant engagement. The second iteration of the installation was displayed at the David Braley Health Sciences Centre at McMaster University (Hamilton, ON), which was coordinated by team members (SI, MH, and SH). This case study details these first two sites only as no further changes were made to the installation, and public input gathered from these sites informed the subsequent qualitative study.

### 2.5 Descriptive Analysis and Informing of Qualitative Research

Public input was analysed descriptively by the research coordinator. Categorical questions were reported as proportions; raw frequencies and percentages were reported to allow comparison across probes, where the number of responses varied. These findings were compared to population‐level quantitative findings (Phase 1); this comparison involves extrapolation levels of analysis, and while recognising the limitations of cross‐level comparison, this approach allowed us to contextualise individual experiences within broader care patterns. Unlike the quantitative findings (Phase 1), which included participants with specific disease diagnoses, the installation open to the general public; as an unattended and anonymous exhibit, it was not designed to attract attendees who were comparable to the Phase 1 research or yield a similarly representative sample. Participants were able to provide brief written responses, which were collated. Findings from the public engagement were reviewed by the team to inform the interview guide for qualitative interviews conducted with bereaved caregivers regarding their experiences of COC at the EOL.

Ethics approval was received for the qualitative interview recruitment components of this project from the Bruyère Health Research Ethics Board (REB) (REB number: M16‐24‐001). The Bruyère Health REB waived the requirement for ethics approval of the design installation as it collected anonymous data with no possibility of linkage to participants (see Supporting Information: File 1 for details).

## 3 Results

### 3.1 Contemplation of Quantitative Findings and Selection of Installation Direction

Examination of the quantitative findings yielded rich discussion of peoples' EOL care experiences in Ontario. The team identified challenges with care coordination and access to specialist and palliative care. The team discussed gaps in the current understanding of EOL care quality and considered questions including ‘what aspects are important for a good death?’, and ‘who is involved with and in charge of EOL care?’.

### 3.2 Development of Design Principles

Design principles developed by the team included: accessibility to a general public audience; engaging with quantitative findings at a conceptual level; aiming to elicit reflection on lived experience over projection of an imagined future; and the prioritisation of responding in tactile ways over writing (Table 1).

**Table 1:** Design principles.

**Our installation will…**	**Definition**	**Considerations discussed**
Aim to be accessible, rather than specific	Focus on engaging the general public. We will assume no healthcare expertise. We will use plain language.	When designing probe, the shorter and broader, the better.
Aim to be conceptual, rather than literal	Strive to be more conceptual, rather than literal. Include broad questions that invite wide participation or interpretation. Use form and imagery that are striking and prompt intrigue.	
Aim for reflection, rather than projection	Balance being a platform for expression and reflection. We will invite participants to draw on their perspectives and feelings (vs. projection into a future or experience they have not had).	How to design a probe about EOL to avoid everyone projecting – we haven't died yet!
Strive for active participation, rather than passive viewing only	We will prioritise participant engagement over sharing knowledge from the research.	
Prioritise responding, rather than writing	We will opt for low barriers to participation like voting, ranking, and reacting to prompts in the installation. This is a lighter lift for participation than asking participants to share their own words, and more conducive in a space with passersby.	Probe needs to collect responses that are synthesizable.

The final installation was effectively guided by our design principles, with two minor adaptations made to strengthen engagement and ensure data synthesizability, while remaining true to the principles' intent. First, while we aimed to leverage reflection over future projection, we recognised that not all participants have experience with EOL healthcare upon which they can reflect. By developing two probes that invited reflection and projection respectively, any member of the general public would be able to relate to the probes and subsequently engage. Second, in balancing our priority of tactility over writing with the constraint of ensuring data was easily synthesizable, we developed a probe that elicited ‘written’ responses (i.e., physically written) in the form of a multiple‐choice form, which did not require long‐form textual responses. We balanced our prioritisation of responding over writing by including an open‐ended probe for participants to include an optional textual comment, and by including embodied engagement in which participants were able to tangibly interact with the exhibit.

### 3.3 Concept Ideation, Selection, and Refinement

Based on contemplation of the quantitative findings and clarification of design principles, the design researchers presented five initial design concepts for consideration (Supporting Information: File 1). Following structured feedback using ‘Rose, Bud, and Thorn’ for each, the team's votes indicated preferences for two concepts, including ‘Language scales’ and ‘Networks (pie style)’.

‘Networks’ was considered appealing in its tactile approach to engagement (i.e., indicating experiences or preferences with coloured pom poms). We developed and considered a second refined iteration of this concept, however, we ultimately did not move forward with this concept due to limitations including: difficulty distinguishing between actual and ideal experiences; inability to visually represent the relative importance of different responses; and logistical challenges in capturing engagement while preserving the physical installation.

‘Language scales’ invited participants to place votes along a scale to indicate their understanding and/or preference for elements of COC. Positive aspects of this concept included the ease of engagement for participants, its alignment with our research focus, and the capacity to highlight various aspects of participant knowledge, impressions, and preferences. This concept aligned strongly with our design principles in the simplicity of the probe and interaction. However, the dichotomous nature of scales restricted participant responses to a binary. The design researchers highlighted the potential difficulty of engagement with elements of quality of care as a non‐expert member of the public. We were thus interested in exploring this topic while facilitating meaningful engagement for the general public. We also sought to create a more compelling probe that would ‘hook’ the public's attention and participation. Incorporating these strengths, limitations, and potential adjustments, the design researchers developed a refined iteration of this concept (Supporting Information: File 1).

**Figure 1:**
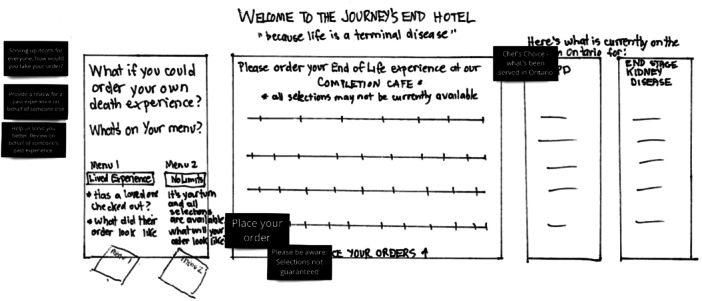
Main display iteration sketch.

Broadening from the dichotomous ‘Language scales’, the ‘Menu for the end’ was inspired by a diner restaurant (Figure 1), in which a paper order form of menu options is provided to participants; however, instead of burger toppings, the form probed about experiences at the EOL (e.g., the involvement of particular physician specialties). Participants would be invited to reflect and complete a paper‐based form, selecting preferences for their ‘order’ of their desired EOL experience. Once completed, participants then placed these forms into restaurant chit holders mounted on the installation surface, mimicking the practices of traditional diner transactions, and allowing the public to see how others would take their orders for EOL. This tangible interaction transformed participants from passive observers into active co‐creators through direct physical manipulation of artifacts within the installation space. The concept was strengthened by the stark and provocative invite, creating a sense of surprise and intrigue surrounding the probe. The humour and juxtaposition of pairing the topic of death alongside the lightness and familiarity of a diner was anticipated to enable this challenging subject to be more accessible to the public. This concept also included a nuanced set of responses that would capture rich data and have the potential to demonstrate patterns in preferences for EOL care. In the team's discussion, we proposed a second version of the order form, that would be called a restaurant ‘review’ that, using a parallel probe, inviting reflection on an actual, lived EOL experience.

**Figure 2:**
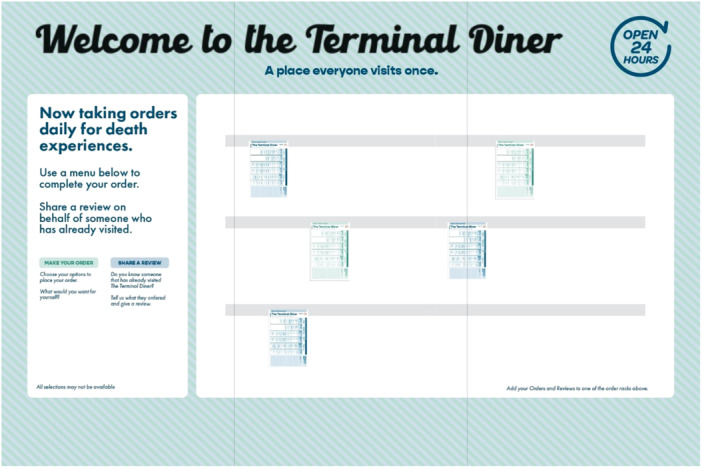
Main display prototype.

‘Menu for the end’ was selected through consensus by the team as the most compelling conceptual direction, due to (1) the novelty and intrigue of the probe and activity, (2) its alignment with our design principles, particularly the tactile mode of engagement, and (3) its capacity to incorporate prior research and produce outputs that could inform subsequent research. The design researchers then developed further iterations of the main installation display and interactive materials (Figure 2) based on team and non‐team member feedback (Supporting Information: File 1).

### 3.4 Installation Prototyping, Production and Iteration

Based on ‘Menu for the end’, the installation was entitled *The Terminal Diner*. Borrowing characteristics of a classic diner, the probe invited the public to anonymously complete orders for their own ideal EOL healthcare experience—using an order form (Figure 3a)—or leave reviews drawn from their lived experience in accompanying a loved one's end‐of‐life journey—using a review form (Figure 3b).

Figure 3(a) First order form iteration. (b) First review form iteration. (c) Second combined order/review form iteration.

**Figure:**
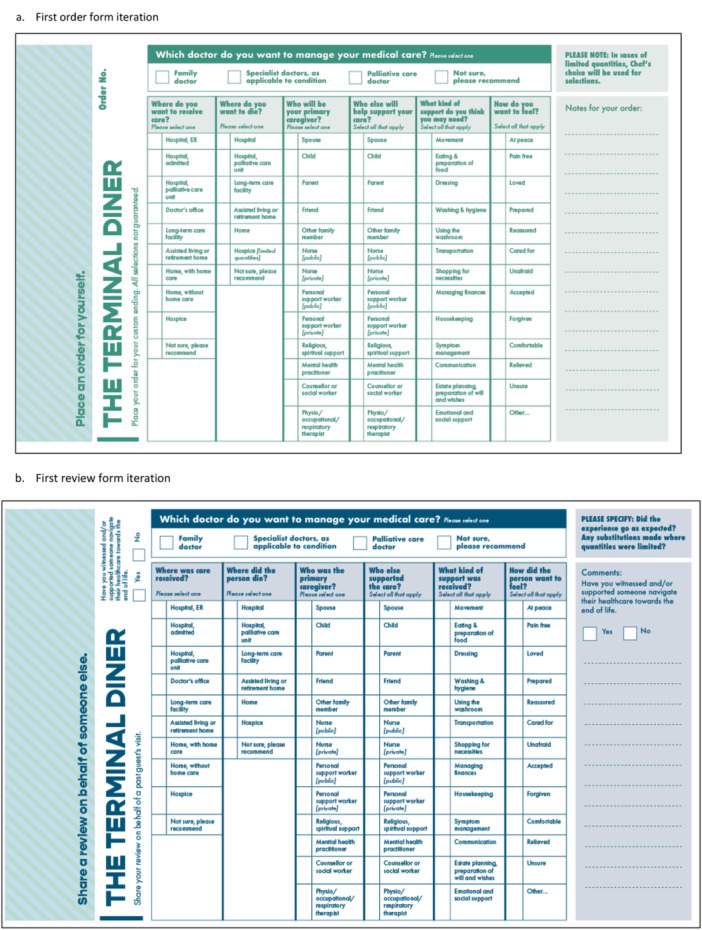

**Figure:**
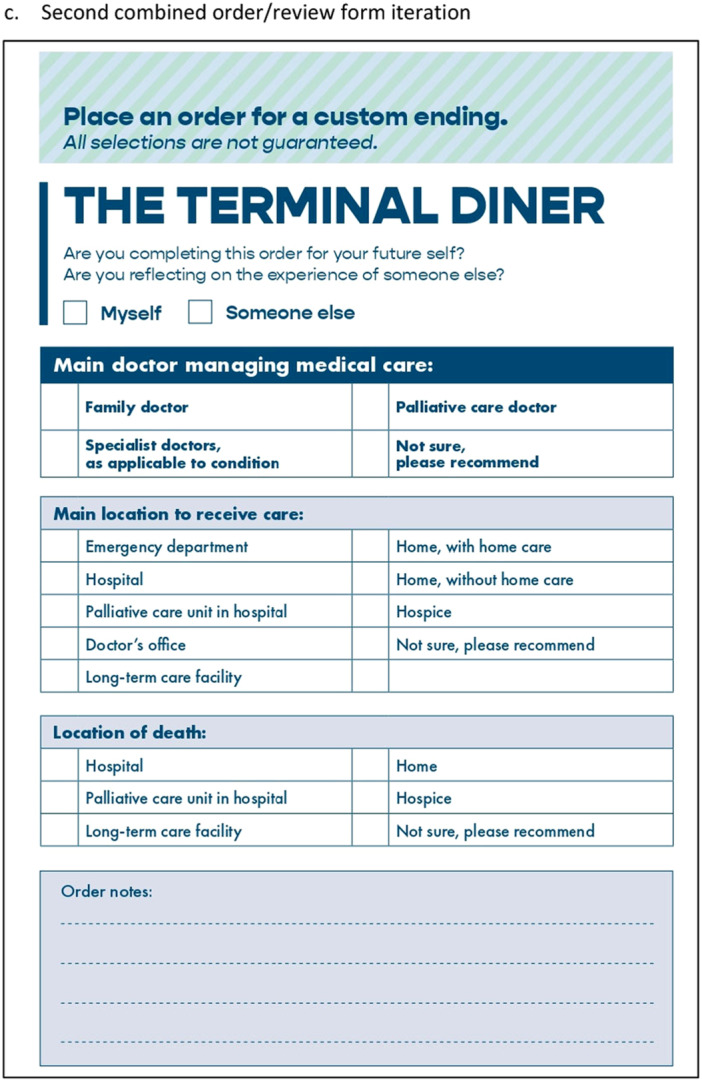

To optimise the installation's aesthetics, our guiding consideration was legibility and familiarity, as we wanted visitors to feel drawn in by recognition. We aimed for it to be striking across exhibition venues while reflective of our design principles and mood board (Supporting Information: File 1). Rather than a literal or nostalgic interpretation of the ‘diner’ theme, we abstracted its elements. A stripe motif referenced familiar diner decor, paired with a contemporary, unornamented typeface to keep the aesthetic clean and approachable. Red accents reinforced the installation's warmth and improved accessibility through visual contrast. Together, these choices aimed for a visual identity that felt familiar but understated to foreground the participatory experience.

**Figure 4:**
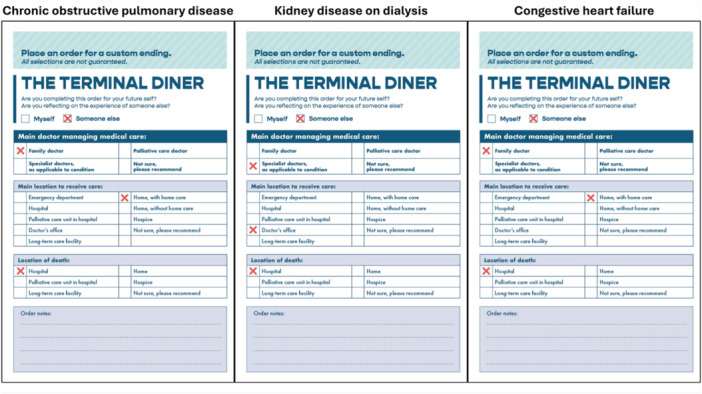
Population‐level reviews completed based on Phase 1 findings.

Production of the installation elements (main display, banner, interactive materials, and recruitment fliers) took place in Toronto, ON. The main display, which included the probes and interactive elements, was printed onto three foamcore boards, which were placed adjacent to one another, attached to three steel stands, and joined together by affixed steel restaurant chit holders. Using a retractable vinyl banner, we displayed three reviews that we completed to reflect EOL care patterns for patients in Ontario, in which selections were informed by the population‐level quantitative findings (Phase 1) of each disease group (KF‐D, COPD, and HF) (Figure 4). The banner also featured a QR code and invitation for those interested to provide their contact information for participation in subsequent qualitative interviews, and recruitment fliers were available on a small freestanding table. The freestanding components of the display measured 81 inches high × 160 inches wide × 30 inches deep. Order forms, review forms, and recruitment fliers were printed and cut, and stored alongside clipboards and pencils in an accompanying rolling cart.

#### 3.4.1 Iterations

**Figure 5:**
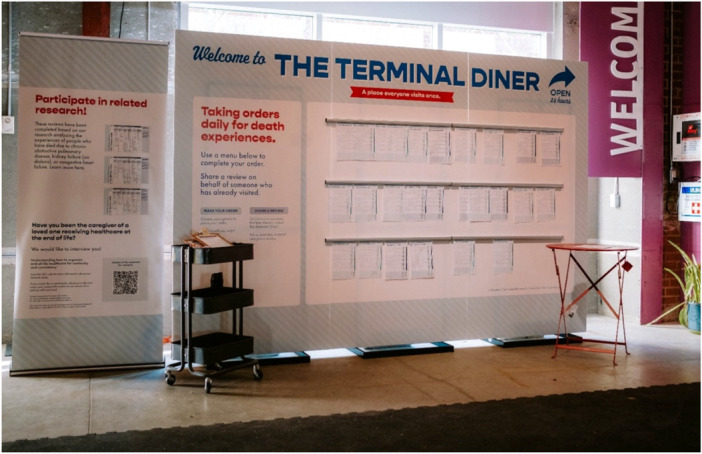
*The Terminal Diner* at Evergreen Brick Works. *Photo Credit:* Karl Everett.

The first iteration of *The Terminal Diner* was displayed at the Evergreen Brick Works for 9 days (27 January 2024–4 February 2024) from 9 AM to 5 PM daily (Figure 5). Brick Works provides immersive experiences of community and sustainability, hosting a variety of programmes including interactive workshops and festivals; *The Terminal Diner* was on display during two DJ Skate Night events and a farmer's market. Open to the public, *The Terminal Diner* was visited by a varied audience including “farmers market foodies, kids, art fans, nature lovers, and well, everyone!” [26]. *The Terminal Diner* was exhibited in the main lobby next to the Visitor's Information desk. We employed several promotional strategies to prompt engagement with the installation, including the development and release of a media advisory shared with colleagues and members of the press. The installation was also promoted on local news and event promotion websites including blogTO, Toronto.com, and Now Play Toronto.

Twenty‐nine order forms (desired experiences) and 23 review forms (actual experiences of a loved one's EOL) were completed by the public at Evergreen Brick Works. Nine individuals consented to be contacted to participate in a qualitative interview. On‐site observations revealed barriers to public engagement with the installation, which the team addressed through iterative changes (Table 2). The second iterations of the order/review forms (Figure 3c) and banner were produced.

**Table 2:** Changes to the installation following observation.

Observed challenges	Changes made to optimise engagement
Colours of order forms and reviews were similar and were difficult to distinguish between, thus posing a challenge to effective engagement.	Combined order & review forms, using same language and tense for all questions Added question: “*Are you completing this order for your future self or reflecting on the experience of someone else?”*.
Order and review forms were formatted as landscape orientation, however, were hung in portrait orientation once completed. This created a barrier to reading the completed forms easily.	Reformatted orientation of order forms to portrait
Order and review forms were dense with questions and selection options resulting in a bigger ‘ask’ of participants, ultimately leading to less accessible engagement.	Condensed questions to include minimum viable information. Included only: *“Main doctor managing medical care”; “Main location to receive care”; and “Location of death”*. Reduced options within each question, and adjusted wording employing more lay language, to maximise understanding. E.g., *“Hospital, admitted”* to “*Hospital*”.
Completed order forms reporting past population‐level findings were difficult to read and engage with due to small font size.	Restructured the banner design to include larger images of order forms with increased font size.
Placement of the recruitment QR code at bottom of banner resulted in difficulty in understanding its purpose and scanning it.	Restructured the banner design to include the QR code and description at eye‐level.
The rolling cart used to hold pencils, clipboards, and order and review forms was easily moved and made inconspicuous, resulting in a barrier to participation.	Clipboards and pencils were attached to installation with hooks and containers.

**Table 3:** Menu and Review form versions.

Version 1	Version 2	Changes
	**Are you completing this order for your future self and what you hope for, or are you reflecting on the experience of someone you supported in navigating their healthcare at end of life?**	Consolidated menu and review forms; added a differentiating question to capture desired versus lived experience
	Myself	
	Someone else	
***Menu*** **/** ***Review*** **: Which doctor do you want to manage your medical care?**	**Main doctor managing medical care:**	
Family doctor	Family doctor	
Specialist doctors, as applicable to condition	Specialist doctors, as applicable to condition	
Palliative care doctor	Palliative care doctor	
Not sure, please recommend	Not sure, please recommend	
***Menu*** **: Where do you want to receive care?** ***Review*** **: Where was care received?**	**Main location to receive care:**	
Hospital, ER	Emergency department	
Hospital, admitted	Hospital	
Hospital, palliative care unit	Palliative care unit in hospital	
Doctor's office	Doctor's office	
Long‐term care facility	Long‐term care facility	
Assisted living or retirement home	—	Treated as “Home, with home care”
Home, with home care	Home, with home care	
Home, without home care	Home, without home care	
Hospice	Hospice	
Not sure, please recommend	Not sure, please recommend	
***Menu:*** **Where do you want to die?** ***Review:*** **Where did the person die?**	**Location of death:**	
Hospital	Hospital	
Hospital, palliative care unit	Palliative care unit in hospital	
Long‐term care facility	Long‐term care facility	
Assisted living or retirement home	—	Treated as “Home”
Home	Home	
Hospice	Hospice	
Not sure, please recommend	Not sure, please recommend	
***Menu:*** **Who will be your primary caregiver?** ***Review:*** **Who was the primary caregiver?**		Removed question
Spouse		
Child		
Parent		
Friend		
Other family member		
Nurse (public)		
Nurse (private)		
Personal support worker (public)		
Personal support worker (private)		
Religious, spiritual support		
Mental health practitioner		
Counsellor or social worker		
Physio/occupational/respiratory therapist		
***Menu:*** **Who else will help support your care?** ***Review:*** **Who else supported the care?**		Removed question
Spouse		
Child		
Parent		
Friend		
Other family member		
Nurse (public)		
Nurse (private)		
Personal support worker (public)		
Personal support worker (private)		
Religious, spiritual support		
Mental health practitioner		
Counsellor or social worker		
Physio/occupational/respiratory therapist		
***Menu:*** **What kind of support do you think you may need?** ***Review:*** **What kind of support was received?**		Removed question
Movement		
Eating & preparation of food		
Dressing		
Washing & hygiene		
Using the washroom		
Transportation		
Shopping for necessities		
Managing finances		
Housekeeping		
Symptom management		
Communication		
Estate planning, preparation of will and wishes		
Emotional and social support		
***Menu:*** **How do you want to feel?** ***Review:*** **How did the person want to feel?**		Removed question
At peace		
Pain free		
Loved		
Prepared		
Reassured		
Cared for		
Unafraid		
Accepted		
Forgiven		
Comfortable		
Relieved		
Unsure		
Other…		
***Menu:*** **Notes for your order** ***Review:*** **Comments**	**Order notes**	
[Free text]	[Free text]	
***Review:*** **Have you witnessed and/or supported someone navigate their healthcare towards the end of life?**		Integrated into the combined menu/review form prompt
Yes		
No		

**Figure 6:**
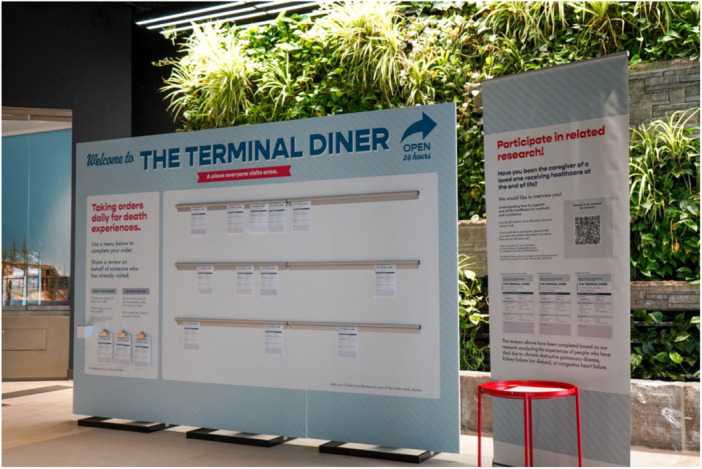
*The Terminal Diner* at David Braley Health Sciences Centre, McMaster University.

The second iteration of the installation was displayed at David Braley Health Sciences Centre for 36 days (24 February 2024–30 April 2024), available to the public from 9 AM to 5 PM on weekdays (Figure 6). This venue is a McMaster University Faculty of Health Sciences building and houses a series of public spaces and informal meeting areas. It is also home to McMaster's Department of Family Medicine, university teaching facilities, clinics, and the City of Hamilton's Public Health Services. *The Terminal Diner* was displayed in one of the facility's public indoor gardens on the main level with visitors often including healthcare workers, patients of maternity, family practice, and public health clinics, as well as McMaster staff and students. Promotion of the installation was conducted by McMaster University, including features in the organisation's newsletter and sharing a media advisory with local media outlets. The co‐PIs (MH and SI) hosted a vernissage to promote further engagement, at which approximately 30 colleagues, friends, and family were in attendance.

A total of 81 order forms (desired experiences) and 22 review forms (actual experiences of a loved one's EOL) were completed by the public at David Braley Health Sciences Centre, and ten individuals consented to be contacted for participation in our interviews. *The Terminal Diner* was featured in the local newspaper, *The Hamilton Spectator*, profiled in the Canadian Institutes of Health Research (CIHR) Faces of Health Research, and the newsletter of CIHR's Institute of Health Services and Policy Research.

### 3.5 Descriptive Analysis and Informing Of Qualitative Research

**Figure 7:**
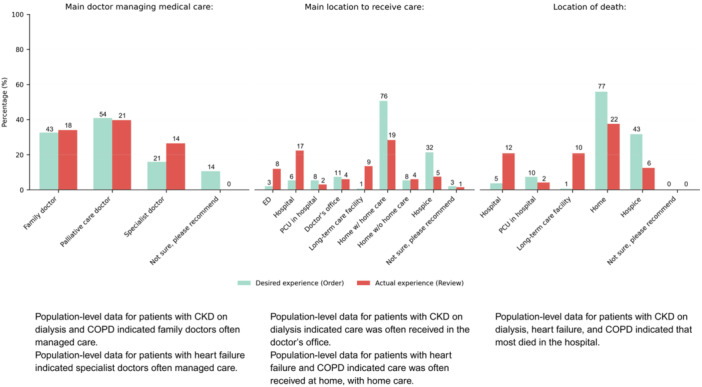
Order and review form selections.

A total of 110 order forms (71.0%) reflecting desired experiences and 45 review forms (29.0%) reflecting actual experiences of a loved one's EOL journey were completed by anonymous members of the general public who engaged with *The Terminal Diner* in Toronto and Hamilton.^1^ Participants’ selections from the primary questions asked across both forms are reported in Figure 7.

A majority of participants placed orders desiring a palliative care physician as their main care provider (49.1%), which was similarly reflected by actual experiences with palliative care physicians reported in reviews (46.7%). A smaller proportion of order forms included care provision by family doctors and specialists than actually experienced according to review forms (difference of 6.7%; 15.6%). The population‐level (based on Phase 1 quantitative findings) indicated that patients with COPD and KF‐D tend to receive majority of care from their family physician, while those with HF receive care from specialists, and while this data is not directly comparable across levels of analysis, the patterns remain consistent with reported experiences.

Participants placed orders demonstrating an overwhelming desire to receive care at home with home care (69.1%), followed by orders desiring care in hospice (29.1%). Reviews reported some care was provided at home with home care (42.2%), which was also experienced according to the population‐level reviews for patients with COPD and HF. However, a greater proportion of care reported in reviews was experienced across other settings, including those that were not desired order selections (i.e., emergency department, hospital, and long‐term care).

Similarly, most participants desired to die at home (70.0%) or in hospice (39.1%) according to the order selections; however, according to reviews, actual deaths were reported to occur in less desired locations such as the hospital (26.7%) and long‐term care (22.2%). This breakdown was reflective of the population‐level reviews in which most patients with KF‐D, COPD, and HF experienced deaths in hospital.

We triangulated the Phase 2 public inputs with quantitative findings (Phase 1) to inform the subsequent phase of qualitative research (Phase 3), comprising interviews with bereaved caregivers. We sought to gain nuanced understanding of our findings to date and ultimately inform the development of a theory of COC at EOL. The initial interview guide had been drafted during the writing of the grant application without consideration of findings from Phases 1 or 2. We updated the interview guide accordingly to narrow in on deceased patients' lived experiences, mirroring the completion of a review form and to probe bereaved caregivers' desires for their own EOL experience, mirroring the completion of an order form; this generated a richer, complex understanding of individual accounts further transforming the anonymous responses we obtained via the design installation. Within the interviews, we added discussion of the population‐level quantitative findings (Phase 1) and design results (Phase 2). We probed participants for comparisons to their lived experience and their interpretations of findings (see Supporting Information: File S2 for comprehensive updates to the interview guide). We then used grounded theory analysis to synthesise findings from all three phases and develop a theory of COC at EOL to enhance our understanding of quality care in this critical period (as described elsewhere [22]).

## 4 Discussion

We developed a novel *research‐to‐public‐to‐research* feedback loop to create a participatory design installation, which in application, enabled the co‐creation of a nuanced understanding of COC at EOL. Using a design‐driven mode of enquiry, we created *The Terminal Diner*, which disseminated quantitative findings, engaged the general public in contemplating and co‐creating around the challenging subject of death—outputs of which were used to inform subsequent qualitative research, while recruiting an interested audience as research participants. In examining the responses captured in the forms, our findings demonstrated that the general public's desired experiences are not often reflected in lived experiences of the deceased (via individual‐ or population‐level reviews).

### 4.1 Methodological Framework: The Research‐to‐Public‐to‐Research Feedback Loop

The originality of the *research‐to‐public‐to‐research* feedback loop arises from the bidirectional meaning making, moving between direct enquiry and creative collective engagement. Leveraging ABKM foundations, this methodology formed a generative epistemic process that yielded insights unique from conventional research alone. This was true in both the process moving between phases—developing the design installation, distiling knowledge from public inputs—and of the public's ‘collective making’ itself, when engaging with the installation.

*The Terminal Diner* highlights the value of effective participatory design in elucidating rich understanding in areas of research that may flatten complex human experience. Throughout the creation process, the design researchers converged on the most effective way to garner participation, iteratively refining the type, means, and purpose for public engagement. Reflection and iteration were critical to enhancing the public's engagement with the installation, thereby drawing them into essential health services research processes, including engaging with previous quantitative research findings, contributing meaningful data, and opting to participate in subsequent qualitative enquiry.

The strengths of the installation rested in the considered choices surrounding the means and motivation for engagement as well as the intrigue produced by its execution, facilitating substantive participation from diverse members of the public in an inherently, otherwise difficult topic. The combination of ABKM, conventional research, and participatory practices inherent to this methodology extended our reach to a broad, inclusive audience; the exhibition sites in Toronto, Hamilton, Ottawa, Montreal, and Victoria engaged diverse audiences who showed comparable participation. Engagement was further facilitated by the installation's pairing of a familiar concept of a ‘diner’ with the subject of death, which ordinarily lies outside the scope of everyday discussion. Along with the cheekiness and humour in its execution, this concept opened the way for the public to explore and engage with death in an accessible manner—our on‐site observations of participants supported this finding.

The conceptual framework we employed aligns with a larger paradigmatic shift towards patient‐ and public‐oriented research and inclusion of individuals with lived experience. Empowered and meaningful involvement of people with lived experience is considered essential to inform “quality, patient‐centred care, which ultimately drives quality improvement” [27]. The *research‐to‐public‐to‐research* feedback loop inherently prioritises this participation, alongside using co‐design methods for the installation itself and engaging people with lived experience (i.e., bereaved caregivers) as interview participants. This final step of knowledge exchange and public consultation completes the novel *research‐to‐public‐to‐research* feedback loop, extending methodologically from our previous *research‐to‐public* feedback loop (employed in our Going Home to Die installation) [16]. To recruit for the final stage of our research, we found the use of a design installation situated within a mixed‐methods study to be effective, in that we were better able to reach and engage those who were both eligible and interested in engaging with the topic of death as people with lived experience. *The Terminal Diner* was ultimately the most effective recruitment strategy for qualitative interviews when compared to conventional strategies we also employed (e.g., recruitment posters in primary care clinics, social media posts shared with bereavement communities).

### 4.2 Empirical Findings: Knowledge Emerging from the Case

The case study findings reveal several novel contributions that we examine in turn. The abstraction of quantitative findings (Phase 1) into design outputs expanded what is typically included in the realm of *research as design* to translate outcomes of statistical analyses. Despite the strengths associated with ABKM, art and design is often situated in the qualitative and mixed‐methods research tradition; there remains little focus on interpreting quantitative findings using art and design [15]. In employing the feedback loop methodology, *The Terminal Diner* effectively disseminated findings that are relevant to the general public in Canada, which may have otherwise remained inaccessible, unclear, or uninteresting to many viewers, and thus relegated to an academic or medical audience.

The installation also played a central and novel role as a medium of enquiry, enabling the quantification of lived experiences (i.e., orders and reviews) using design methods. As a result, we obtained a collection of responses that enabled direct comparisons between population‐level actual (Phase 1 quantitative findings), individual actual (Phase 2 reviews), and individual desired EOL care experiences (Phase 2 orders)—the differences between which emerged as meaningful and generative. Our method of comparison thus subverts the well‐established hierarchy of evidence (i.e., quantitative methods as providing the ‘best evidence’) [28]. This method instead foregrounds insights that capture nuance, lived experience, and complexity that are often limited in purely quantitative approaches. Situating quantitative findings within the feedback loop enabled examination of the internal validity of the findings, exploration of the context in which those findings exist, and their ecological validity [29].

The direct comparison of data from three distinct sources (i.e., abstracted quantitative findings, order forms, and review forms) provided a comprehensive understanding of EOL healthcare experiences in Ontario. This triangulation across data sources provides context and complementary perspectives on these care patterns, while recognising the analytical limitations of this cross‐level comparison. Most lived experiences reported in the public's review forms mirrored the population‐level reviews, however disparities emerged when further comparing the public's desired experiences to actual experiences reported at both the individual and population level. Many participants desired palliative care involvement; however, this was often not the experience as indicated by the individual‐ and population‐level reviews. Canada experiences more barriers to accessing palliative care than other developed countries [30]. Health human resources, amongst other system‐level characteristics have been identified as essential for ensuring access to palliative care. The disparity in the receipt of palliative care may thus represent the tension between the positive public perception of EOL care and scarcity of health human resources in Canada [31]. The data was most aligned when considering the location of care—a majority of desired or actual experiences were reported to occur in an outpatient setting. This finding reflects an overarching shift in healthcare provision from acute, hospital‐based settings to community or outpatient care [32]. Despite the desire of many individuals to die at home and an increasing proportion of deaths now occurring outside of the hospital in Canada [33], actual EOL experiences indicated most still died in hospital. Canadians continue to experience barriers in accessing public and community infrastructure often due to a lack of availability and resources [34]. The various forms of data abstracted as design outputs enabled novel and meaningful comparisons to inform our future research.

## 5 Limitations

We faced several challenges requiring consideration for future design‐driven, EOL research. The physical construction of the installation required deliberate planning with regards to durability and transportability of the materials; the installation was limited by the longevity of its materials and our capacity to transport it. The installation's transportation, assembly and disassembly, and storage added complexity to our study coordination when compared to conventional research. Further, *The Terminal Diner* was designed for unfacilitated engagement which posed several limitations. Firstly, we selected exhibit sites based on their capacity to provide adequate security and periodic monitoring. Secondly, the installation may have elicited less engagement because it was unattended; however, we sought to ensure sufficient facilitation existed inherently in the installation to support participation, while maintaining the autonomy and anonymity of individuals to share and explore this sensitive topic in a public space. Further, without facilitation, we were limited in verifying whether participants noticed or engaged with the reviews reflecting the quantitative findings, so while the installation successfully elicited and displayed public reviews, the extent to which our previous findings were processed by participants remains unknown. Finally, stigma surrounding the topic of EOL and the manner in which it should be discussed remains prevalent. While this methodology created a highly accessible, relatable way to engage with this topic, it is likely that stigma surrounding any discussion of death posed a barrier for some individuals' engagement with *The Terminal Diner*. The cheekiness of our approach also brought levity to a topic of great gravitas—it is therefore likely that those to whom this strategy was not appealing likely would not engage with the installation.

## 6 Conclusion

*The Terminal Diner* represents a new paradigm for conducting health services research using the *research‐to‐public‐to‐research* feedback loop methodology. The methodological process described here elicited experiential, iterative, embodied ways of knowing both through installation development and in the contributions from the public. This methodology further enabled us to recruit across Canada and reach participants with meaningful interest in engaging with the subject of death. We have continued to build on this work in the field of EOL care [5, 35], and our future projects will focus on further application and refinement of this methodology. The potential of this approach also reaches beyond EOL research and can serve as a powerful tool wherever complex or nuanced concepts need to be richly understood. Extending the application of the *research‐to‐public‐to‐research* feedback loop methodology to other research areas represents a worthwhile future pursuit.

## Author Contributions

**Aria Wills:** writing – original draft, writing – review and editing, project administration, formal analysis, visualisation, data curation, investigation. **Kate Wilkes:** investigation, visualisation, writing – review and editing. **Karen Oikonen:** investigation, visualisation, writing – review and editing. **Nyanna Flynn:** investigation, validation, writing – review and editing. **Shuaib Hafid:** investigation, conceptualisation, writing – review and editing, visualisation, formal analysis. **Aleisha Fernandes:** investigation, writing – review and editing. **Michelle Howard:** writing – review and editing, conceptualisation, methodology, investigation, supervision, funding acquisition, resources. **Sarina R Isenberg:** conceptualisation, methodology, funding acquisition, writing – review and editing, investigation, supervision, resources.

## Ethics Statement

Ethics approval was received for the qualitative interview recruitment components of this project from the Bruyère Health Research Ethics Board (REB) (REB number: M16‐24‐001). The Bruyère Health REB waived the requirement for ethics approval of the design installation as it collected anonymous data with no possibility of linkage to participants.

## Consent

Participants provided implicit consent to participate when choosing to engage anonymously with the installation. In addition, we included the following statement on the display: “Your selections are completely anonymous and will be used to help inform our future research on patterns of care at the end of life”.

## Conflicts of Interest

The authors declare no conflicts of interest.

## Supporting information

Supporting File 1

Supporting File 2

## Data Availability

The authors have nothing to report.

## Endnotes

1 Although the order and review forms differed between sites, we were able to map responses from the earlier version to the revised version as most changes involved basic rewording and collapsing of categories (Table 3). We have presented results as percentages to enable comparison of selections between differing numbers of order and review forms.
