# Supplementary material for: The Terminal Diner: Serving up a Novel Knowledge Exchange Methodology via Participatory Design Installation
Source: Health Expect. 2026 Jun 23;29(3):e70688. doi: 10.1111/hex.70688 (PMC13291436; doi:10.1111/hex.70688)
Supplement: Supplementary file 1 — Supporting File 1 [file HEX-29-e70688-s002.docx]

### **Supplementary file 1 – Methods appendix**

### A. Preliminary concept selection boards

**Figure A1.** How to read a concept board


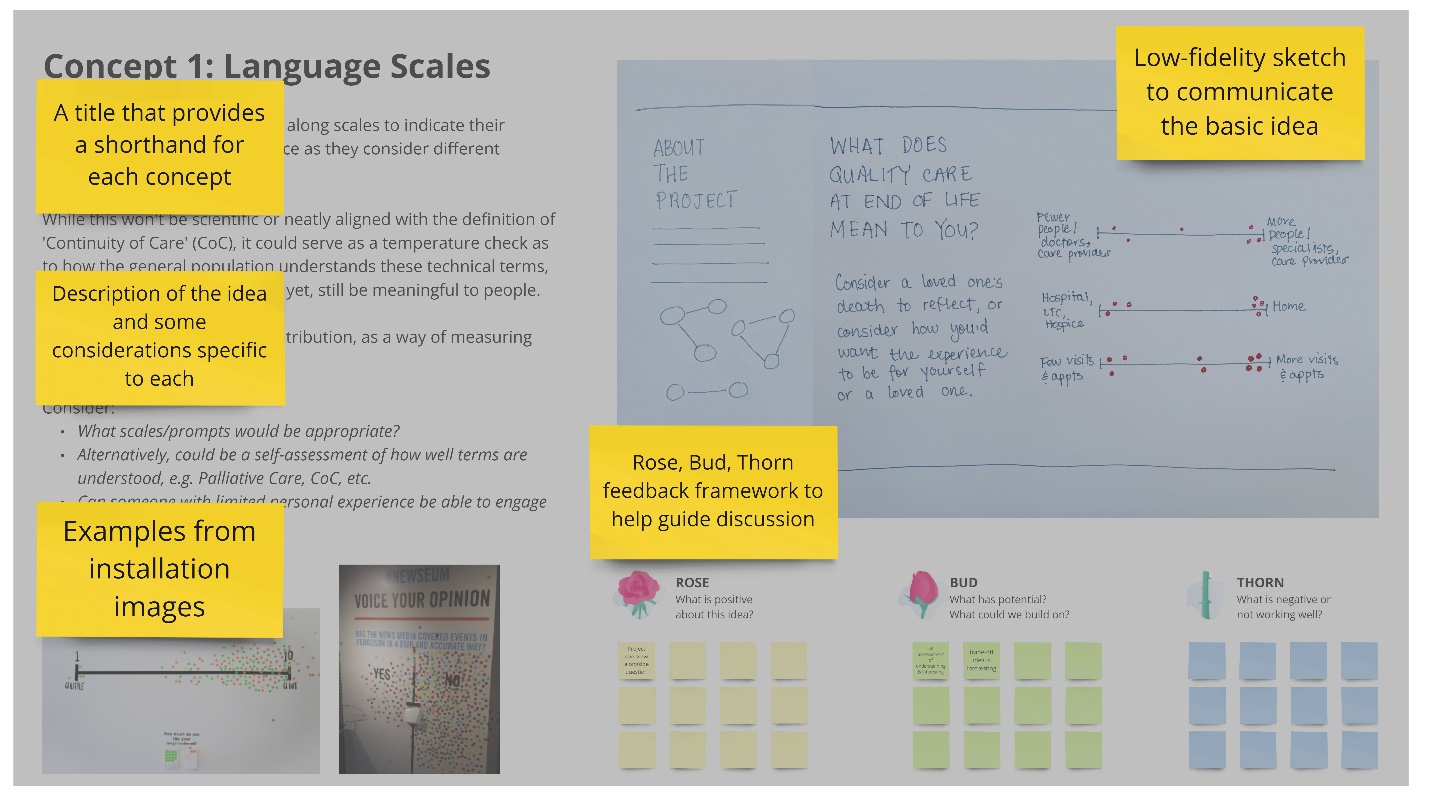


**Figure A2.**
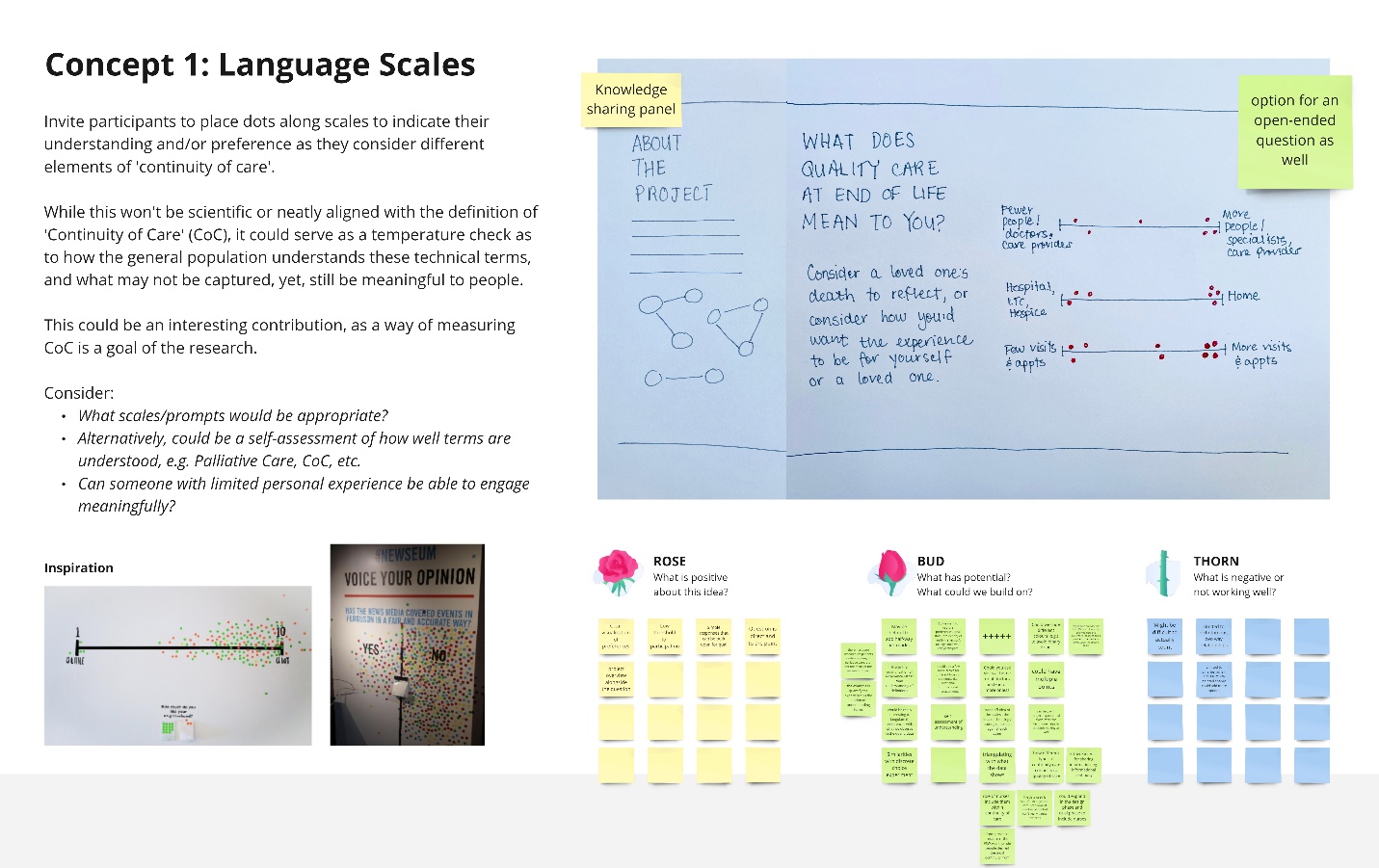


**Figure A3.**
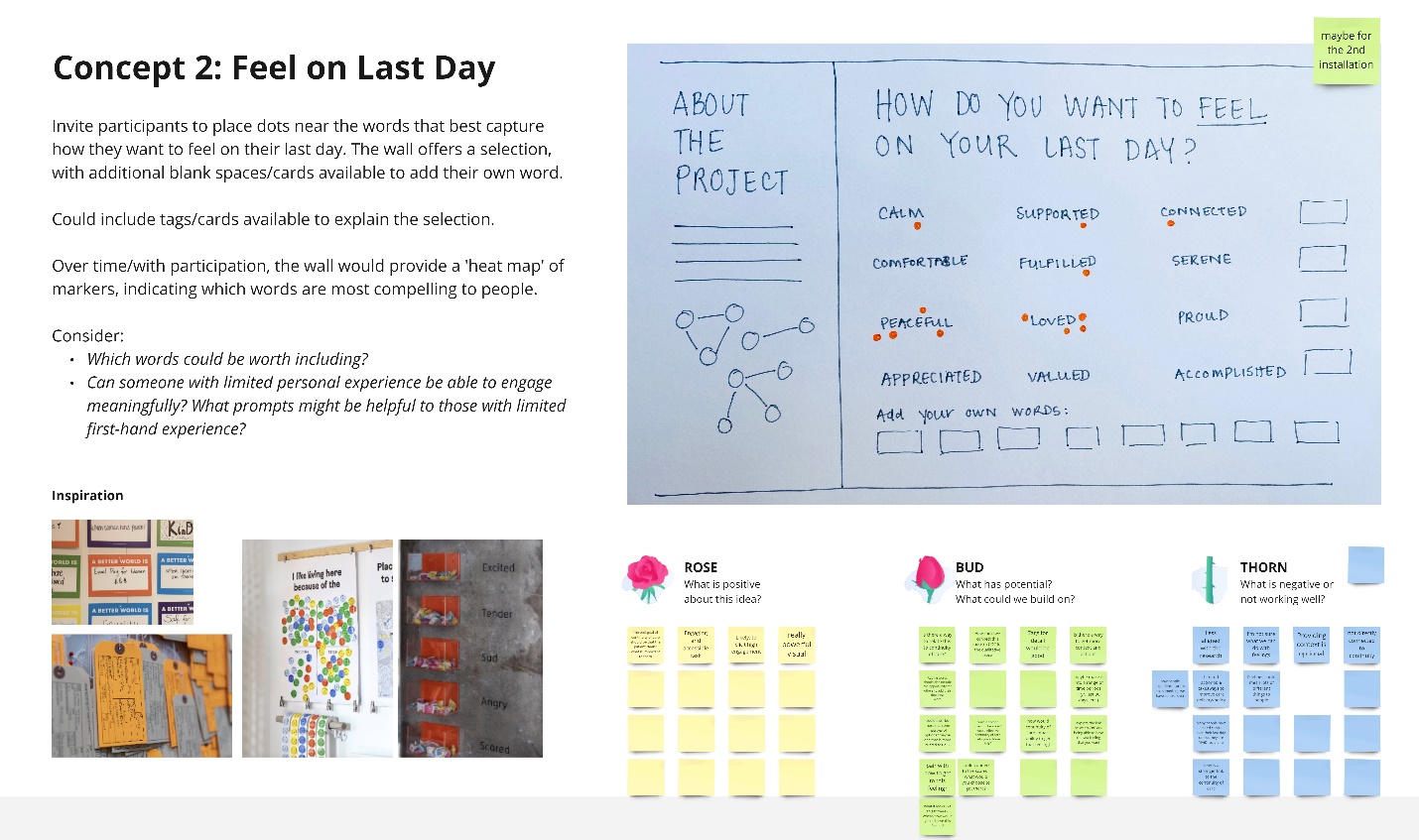


**Figure A4.**
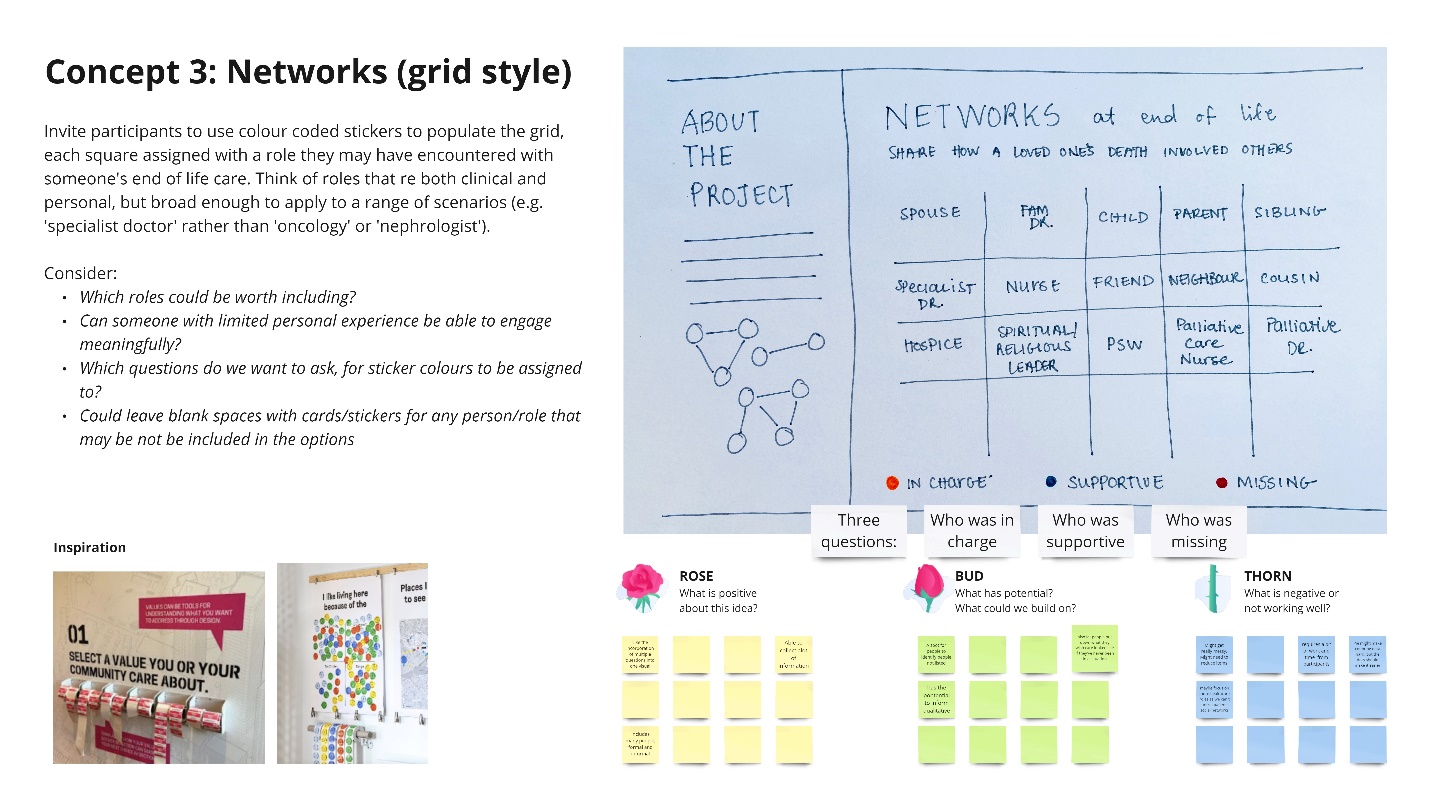


**Figure A5.**


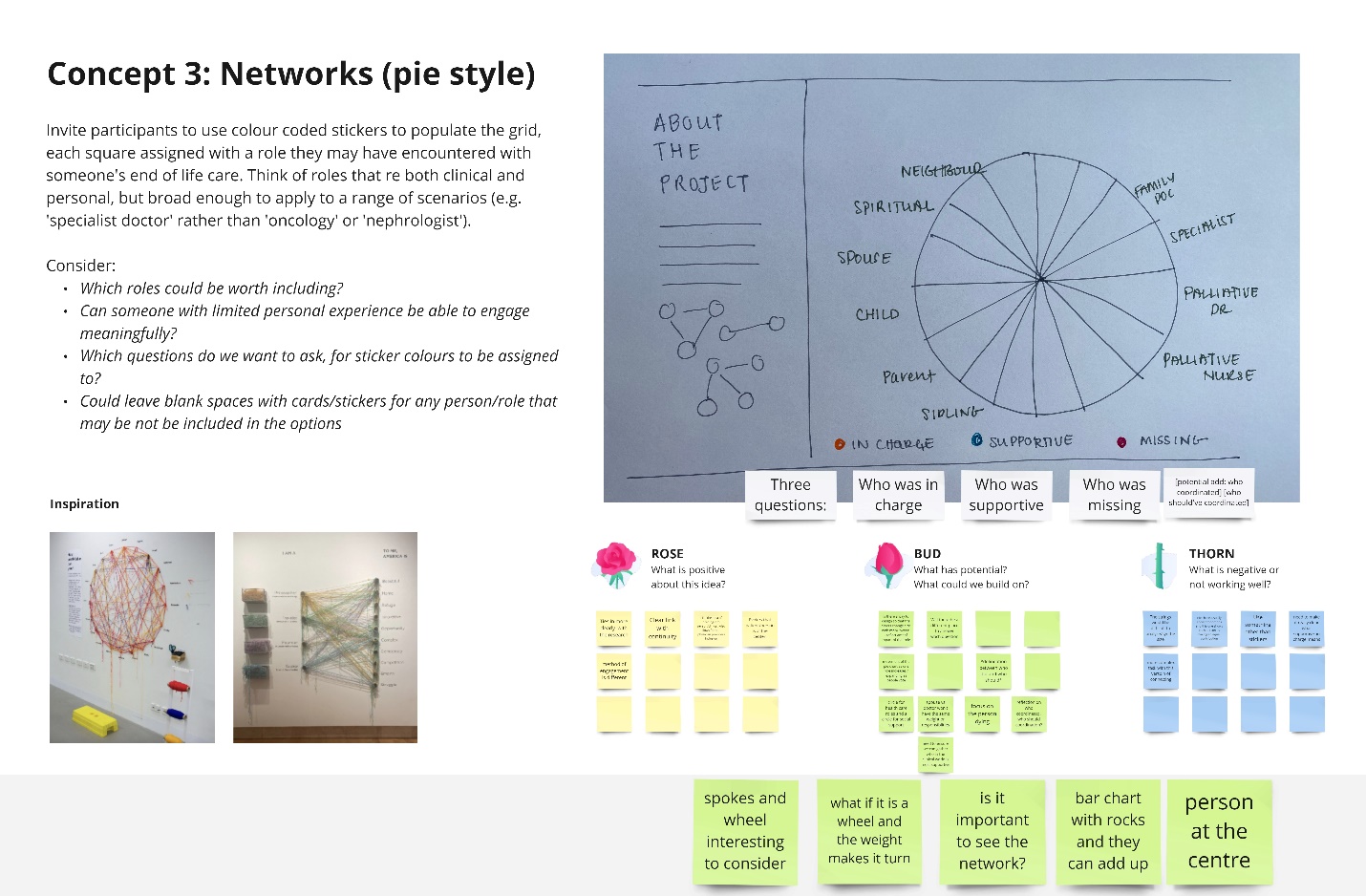


**Figure A6.**
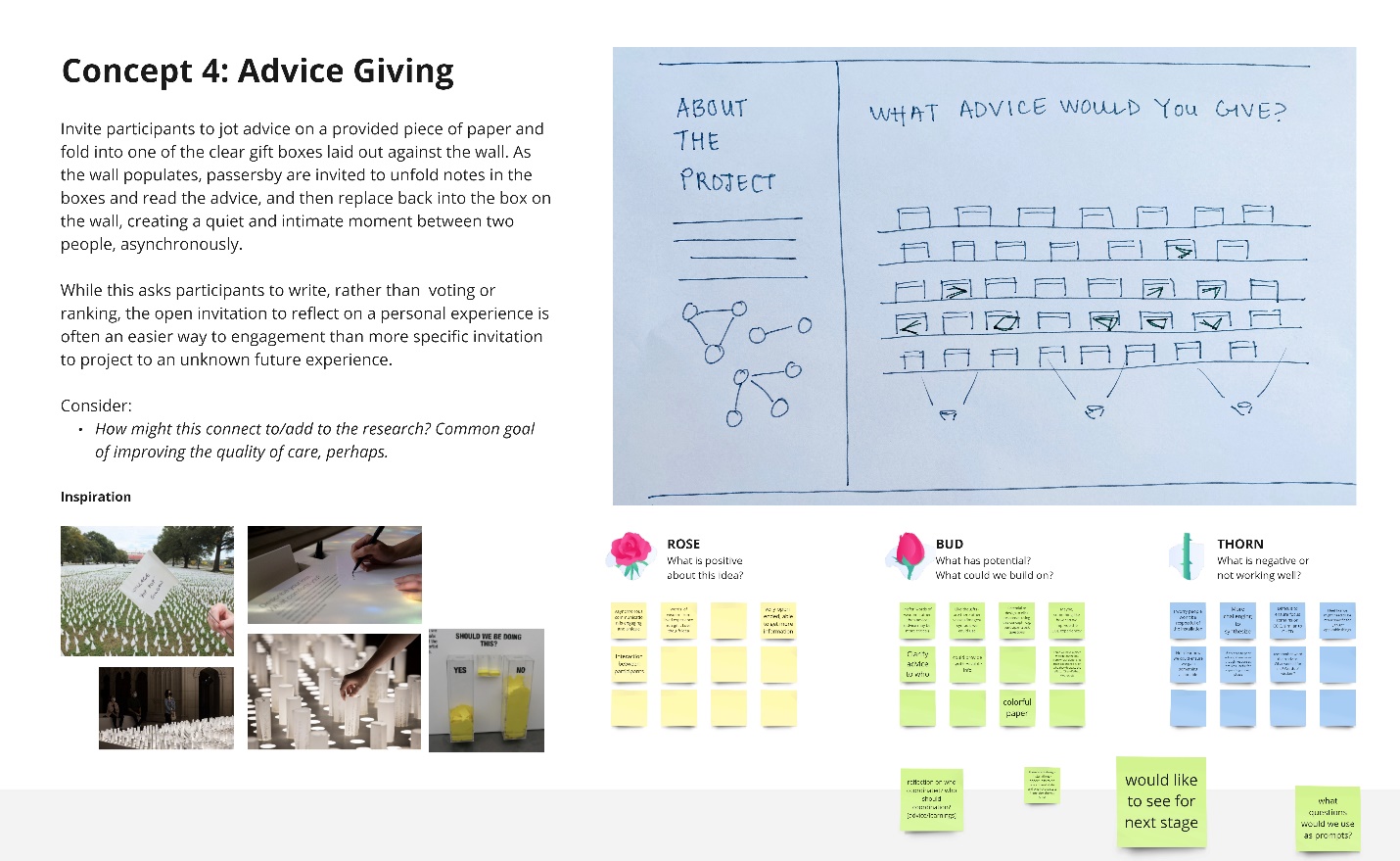


### B. Final concept selection boards

**Figure B1. ‘**Menu for the end’ boards


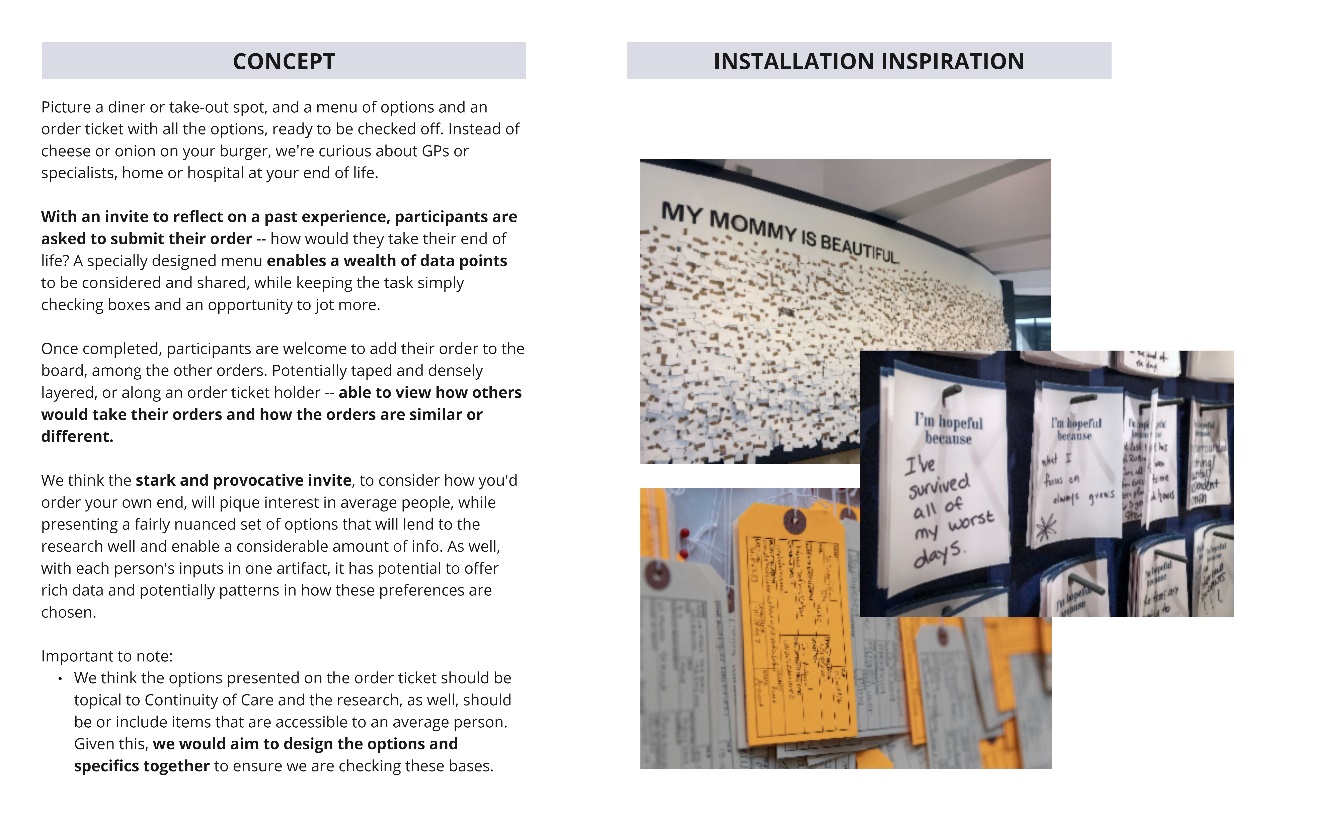


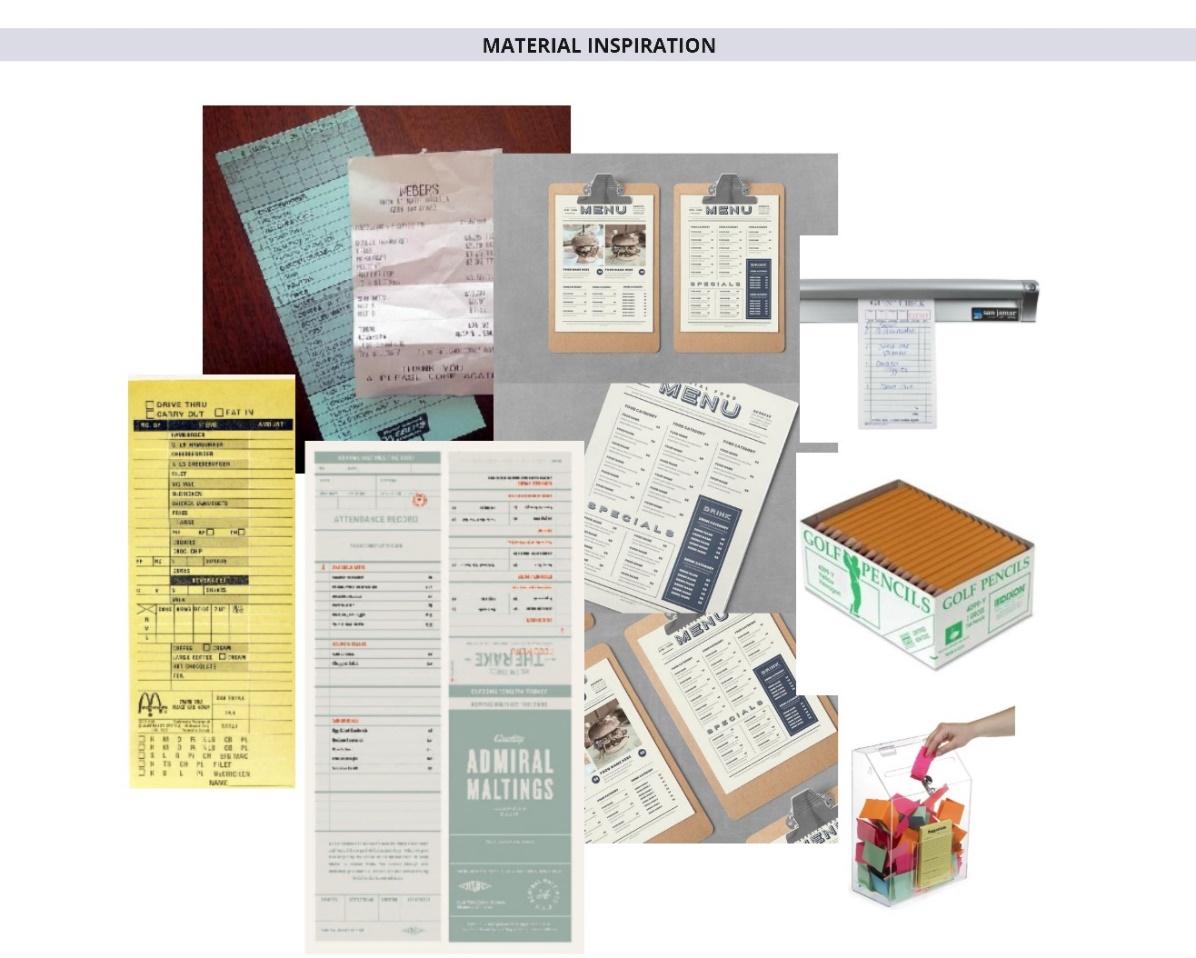


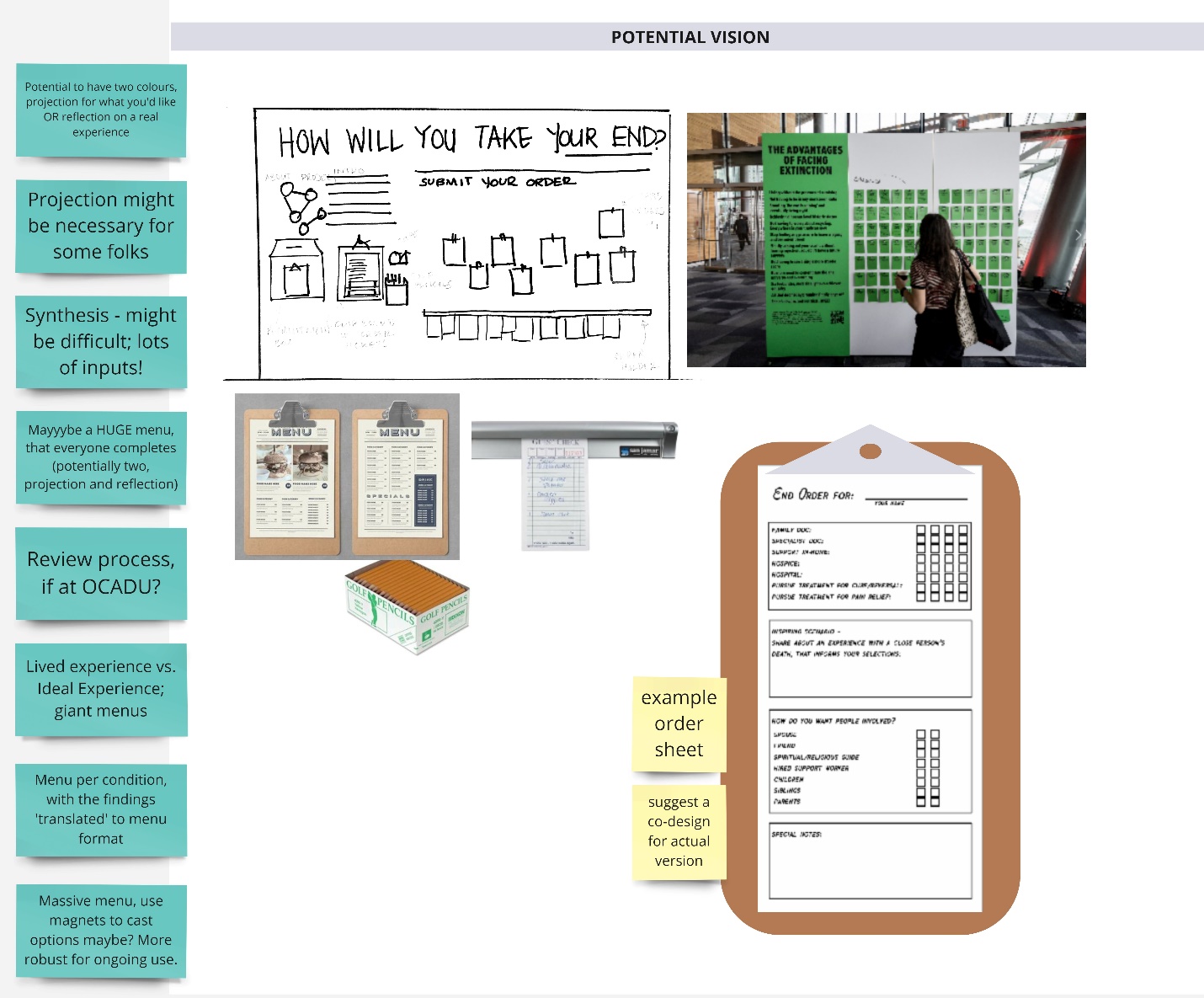


**Figure B2.** ‘Casting ballots’ boards


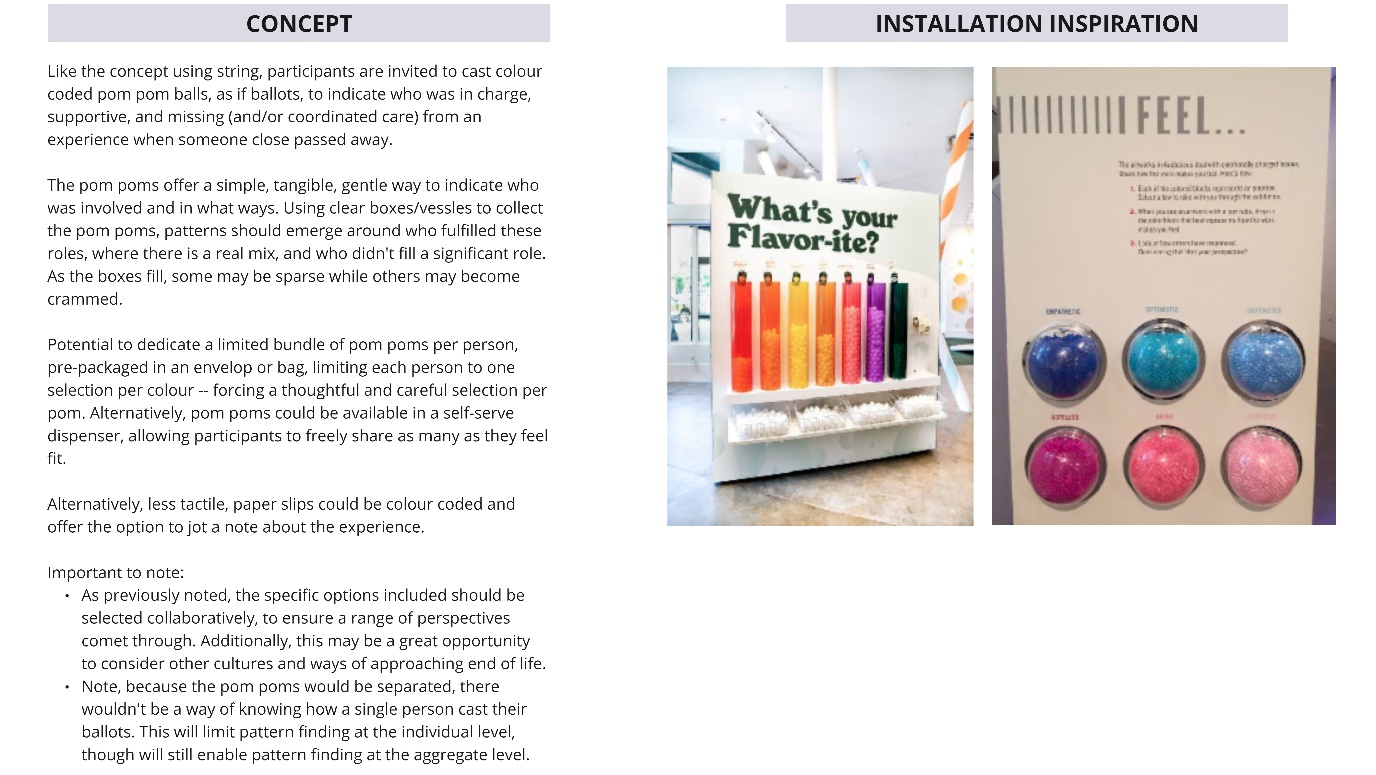


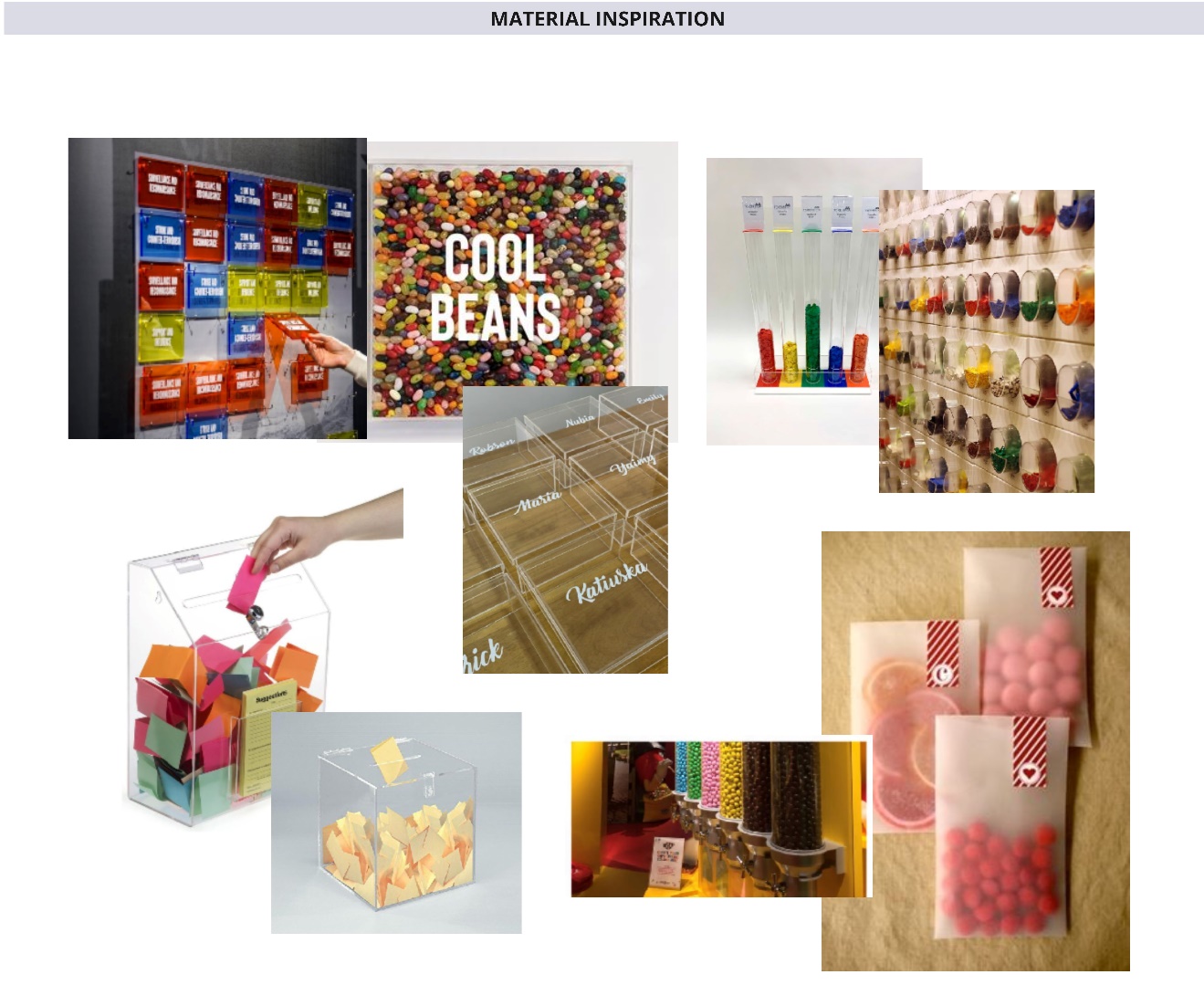


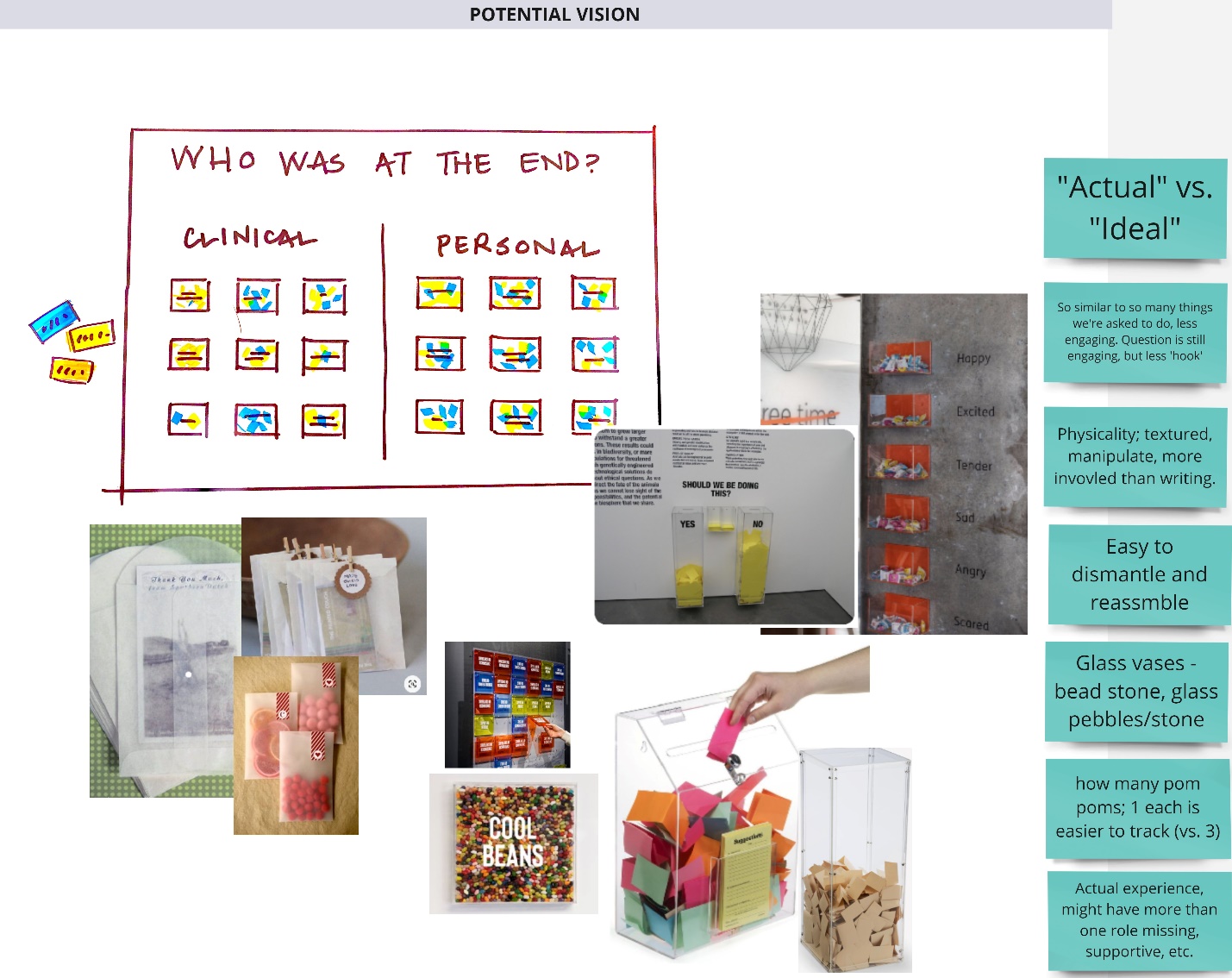


### C. Table – Family & Friend Prototype Feedback

| **Questions to elicit feedback** | **Response themes & areas for improvement** |
| --- | --- |
| What is your overall feedback? | - Unclear wording of questions or order form options - Unclear instructions (e.g., Number of selections, special instructions section, chef’s choice section) - Confusion surrounding design elements (e.g., two ‘Other’ options) - Aesthetic suggestions (e.g., spacing) |
| What is your understanding of the questions and options being asked? | - Aligned understanding of practical asks of probe   - E.g., “I'm being asked to select either one option or all that apply in the top row and then for each column based on my preferences for how I want to die.” - Aligned understanding of relationship between ‘diner’ concept and the probes   - E.g., “They're asking me to select my preferences about EOL care as if I'm ordering food. The menu format seems to be emphasizing or possibly satirizing the fact that making these decisions is not as easy as ordering sushi or a burrito and requires a lot more thought, counseling, discussion with family, context, etc. -- but perhaps that patients are sometimes expected to know these answers. The disclaimers on the upper right and along the left side about choices not being guaranteed in cases of limited quantity suggests that we can't control as much about our death experiences as we may want, and in some cases this may be a problem with a lack of palliative care/medical/ physician resources being allocated to these patients.” - Some contemplation of meaning beyond intended scope of probe   - E.g., “The care team wants to know my preferences and needs on key aspects of my care and what arrangements have already been made for my care so the care team can support me as best they can (within their limits) and probably to help them decide how best to utilize their resources to accommodate me and others as best they can. I may not get everything I want.”   - E.g., “I am being asked to consider my EOL planning. The questions themselves are educational in that they force me to consider the practical implications of what EOL planning involves.” |
| What is your understanding of the task that the exhibit viewer is being asked to do? | - Various understandings surrounding who will receive the ‘order’   - E.g., “I am being asked to give direction to … someone … about my choices for my dying process. I don’t know who I am giving direction to, however.”   - E.g., “Help the care team support me as best they can and make decisions about how to best utilize their resources to accommodate me and others with terminal illnesses as best they can.” - Extensions of understanding to broad implications of engaging with the task, personally and generally   - E.g., “It may also force me to put my mind to these important questions and what I want on key aspects of my care and to make certain necessary arrangements. This will help me, those I want helping me, and help the care team.”   - E.g., “I think the whole point of the exercise is to get me to pause and reflect on what I think would be the ideal way of living before I die. Most people don't do this, so it's a simple way to bring to attention the types of options one should consider, even if sometimes it might feel like we might not have the luxury of choosing all of these aspects. It also brings empathy to people currently in palliative care and what types of support they could use and how nice that would be to give options.” |

### D. Design mood board


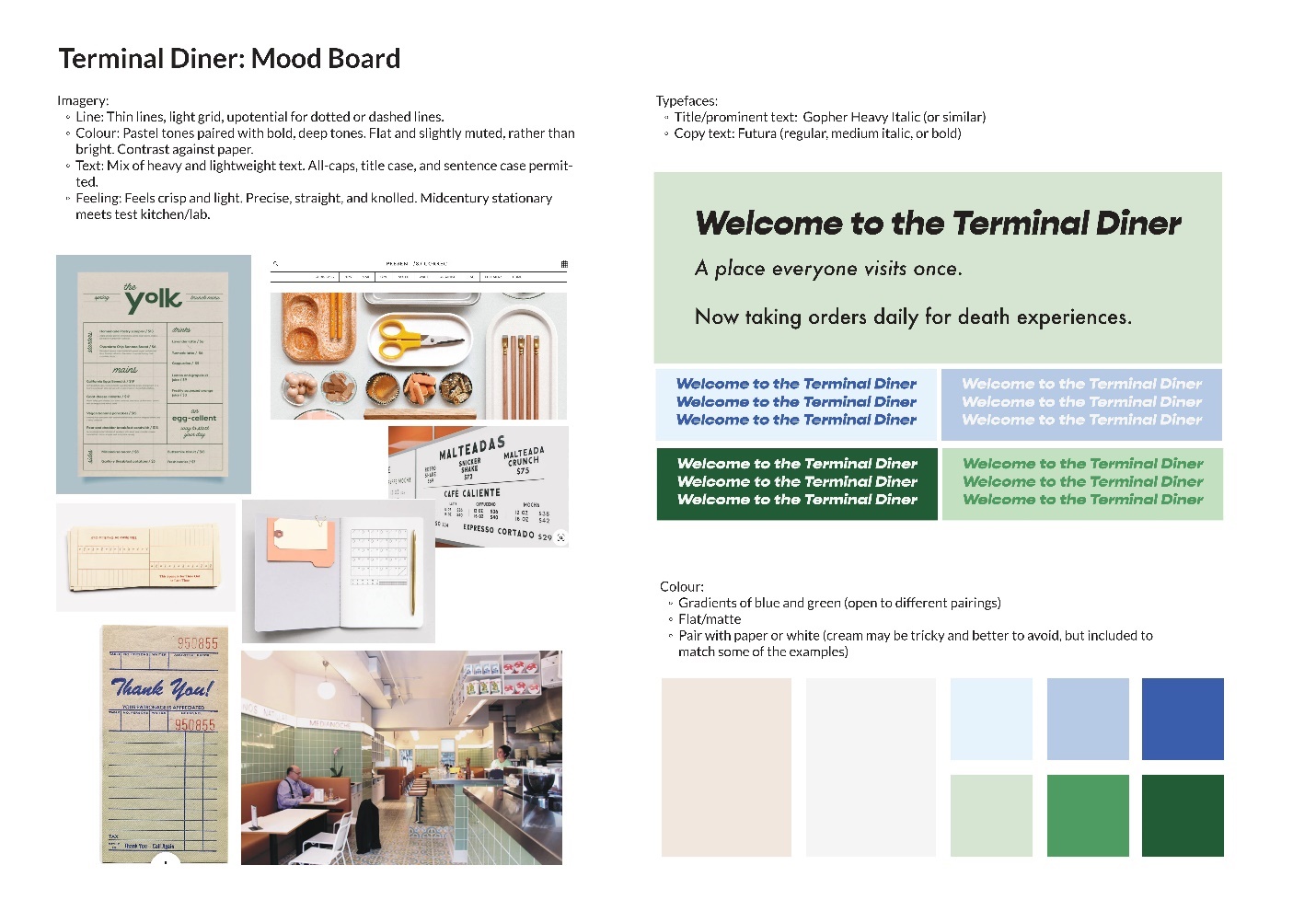


### E. Table – Summary of meetings

| **Meeting number** | **Process stage** | **Meeting focus** |
| --- | --- | --- |
| 1 | Contemplation of quantitative findings, selection of installation direction | **Health services researchers:**  Presentation of summary quantitative findings to design researchers |
| 2 | Contemplation of quantitative findings, selection of installation direction | **Health services researchers:**  Presentation of summary quantitative findings to design researchers and patient/family caregiver advisors  **Team**:  Reflection on findings  Discussion of potential installation themes and questions  **Design Researchers:**  Discussion of physical installation limitations  Presented participatory design with various means of engagement  **Team:**  Reflection on physical installation possibilities |
| 3 | Development of design principles  Concept ideation, selection, and refinement | **Design researchers:**  Presentation of design principles synthesized from previous conversation for team consensus  **Team**:  Reflection on and refinement of design principles  **Design researchers:**  Presentation of five initial installation concepts for team contemplation (**Supplementary File 1 – A. Preliminary concept selection boards**)  **Team**:  Discussion of strengths, challenges, areas for growth using rose-bud-thorn exercise for feedback |
| 4 | Concept ideation, selection, and refinement | **Design researchers:**  Review of initial installation concepts  Presentation of two evolved installation concepts (**Supplementary File 1 – B. Final concept selection boards**)  **Team:**  Discussion of strengths, challenges, areas for growth using rose-bud-thorn exercise for feedback  Poll on preferred installation concept |
| 5 | Concept ideation, selection, and refinement | **Design researchers:**  Presentation of evolved installation concept (**Appendix A. Figure 1**)  **Team:**  Discussion of artifacts & elements regarding content and aesthetic |
| 6 | Installation prototyping, production, and iteration | **Design researchers:**  Presentation of initial installation mock-up  **Team:**  Provision of feedback regarding installation participatory elements, structure, and content |
| 7 | Installation prototyping, production, and iteration | **Design researchers:**  Presentation of prototypes of installation  **Team:**  Provision of feedback regarding installation content |
| 8 | Installation prototyping, production, and iteration | **Design researchers:**  Presentation of iterated prototypes  **Team:**  Provision of feedback regarding installation content and aesthetic |
| 9 | Installation prototyping, production, and iteration | **Design researchers:**  Presentation of iterated prototypes  **Team:**  Provision of feedback regarding installation content and aesthetic |
| 10 | Installation prototyping, production, and iteration | **Team:**  Review of challenges from initial exhibition, brainstorming and planning revisions for second exhibition |

### F. Informed consent process

Engagement with the participatory design installation was reviewed by the Bruyère Health Research Ethics Board (REB) and exempt from requiring approval, as the installation was unmonitored and all responses were anonymous, posing very minimal risk of participant identification. The installation included the text: “Your selections are completely anonymous and will be used to help inform our future research on patterns of care at the end of life.” Participants were understood to have provided implicit consent by completing a menu or review form and affixing it to the installation.

Engagement with the qualitative interview recruitment materials (installation banner and fliers) required approval from the Bruyère Health REB (REB number: M16-24-001). Individuals were considered to have provided implicit consent by engaging with the banner or flyers, both of which featured a QR code linking to a Consent to be Contacted form. Upon completion, a research coordinator would follow up to obtain full informed consent. Recruitment materials included the REB name and number, as well as contact information for the principal investigators. The recruitment fliers also included the study name, description, eligibility criteria, participation requirements, compensation information, and information regarding the right to withdraw.
