# Supplementary material for: The Terminal Diner: Serving up a Novel Knowledge Exchange Methodology via Participatory Design Installation
Source: Health Expect. 2026 Jun 23;29(3):e70688. doi: 10.1111/hex.70688 (PMC13291436; doi:10.1111/hex.70688)
Supplement: Supplementary file 2 — Supporting File 2 [file HEX-29-e70688-s001.docx]

**Supplementary File:** Semi-Structured Interview Guide for Bereaved Caregivers

All changes to interview guide drafted prior to quantitative and design phase, and informed by subsequent findings are highlighted below. Additions to the interview guide are coded in green and changes to existing questions are coded in red.

**Background**

- What was your relationship to the deceased?
  - How long did you serve as a caregiver to the deceased?
  - *Probe: Support with feeding, dressing, transportation, appointments & scheduling, etc.*

**Patient Experience**

In the last year of [patient]’s life:

1. Which type of doctor mainly managed [patient]’s medical care?
   1. *Probe: Family doctor, palliative care doctor, specialist doctor*
2. What other doctors were involved?
   1. How did the doctor’s involvement in their care change over time? [For each doctor mentioned]
3. What other types of medical professionals were involved in [patient]’s care?
   1. *Probe: Nurse, PSW, mental health practitioner, counselor, social worker, PT, OT, RT, spiritual/religious support, death doula*
4. What settings did [patient] receive health care in the last year of life?
   1. How long were [patient] in each setting?
   2. *Probe: Emergency department, hospital, palliative care unit in hospital, doctor’s office, long-term care facility, home with home care, home without home care, hospice*
5. Where did [patient] die?
   1. *Probe: Hospital, palliative care unit in hospital, long-term care facility, home, hospice*

**Caregiver Desired Experience**

1. Which type of doctor would you want to manage your medical care at end of life? What is the reasoning for that?
   1. *Probe: Family doctor, palliative care doctor, specialist doctor*
2. What other doctors would you want involved? What is the reasoning for that?
3. What other types of medical professionals would you want involved? What is the reason?
   1. *Probe: Nurse, PSW, mental health practitioner, counselor, social worker, PT, OT, RT, spiritual/religious support, death doula*
4. What setting(s) would you like to receive health care in during the last three months of life? What is the reason?
   1. *Probe: Emergency department, hospital, palliative care unit in hospital, doctor’s office, long-term care facility, home with home care, home without home care, hospice*
5. Where would you want to die? What is the reason?
   1. *Probe: Hospital, palliative care unit in hospital, long-term care facility, home, hospice*

**Sharing Results**

We previously studied continuity of care at end of life for patients in Ontario with 3 different diseases: chronic kidney disease on dialysis, chronic heart failure, and chronic obstructive pulmonary disorder. We found that family doctors are consistently involved in the care for a majority of patients with heart failure and COPD and involved to lesser extent for patients with kidney disease. Those with kidney disease predominantly received care from nephrologists. Palliative care involvement tended to be concentrated near the end of life, and those who received palliative care experienced fewer deaths in the hospital. Patients with heart failure and COPD predominantly received care at home with home care, while those with kidney failure received care in the doctor’s office. All three types of patients largely died in hospital.

1. How do these results differ from [patient]’s experience or your own desired experience? Why do you think there might be a discrepancy?
2. Do you have any other reflections on these results?

[Using our “Terminal Diner” installation,] we previously asked the general public with lived experience in supporting a patient at end of life about the care the patient received. We found that many individuals described that most patients’ care was received from a family doctor, that many patients received care when admitted to the hospital, and patients tended to die across various settings. We have also asked the general public about their own desired experiences for end-of-life care, and many indicated that they wished to receive end-of-life care from a palliative care physician, at home with homecare, and most wanted to die at home.

1. How do these results differ from [patient]’s experience or your own desired experience? Why do you think there might be a discrepancy?
2. What is the ideal model for end-of-life care including primary provider, setting, location of death?
3. What changes need to happen within our healthcare system to achieve that?
4. Do you have any other reflections on these results?

**Continuity of Care**

As a reminder, we are interested in the idea of 'continuity of care’. Generally, this means things like seeing the same healthcare provider or team over time, knowing that the patient's information is coordinated, knowing that everyone is on the same page.

1. Did [patient]’s experiences of continuity of care vary by doctor?
   1. Probe: *Family doctor, specialist doctor, palliative care doctor*
2. Did their experience of continuity of care vary by healthcare setting?
   1. *Probe: Emergency department, hospital, palliative care unit in hospital, doctor’s office, long-term care facility, home with home care, home without home care, hospice*
3. Can you think of ways that this continuity or discontinuity of care influenced the end-of-life journey of [patient] (e.g. ending up in hospital versus staying at home?)
   1. *Probe: Time spent at home and home supports received, acute care use (e.g., time in emergency room, hospital)*
4. What aspects of the continuity of care were helpful?
5. What aspects of the continuity of care were harmful?

**Wrap Up**

1. Is there anything else you would like people to know about continuity and discontinuity of care at the end of life?
